# ﻿A new species of *Pristimantis* (Amphibia, Anura, Strabomantidae) from a montane forest of the Pui Pui Protected Forest in central Peru

**DOI:** 10.3897/zookeys.1219.129773

**Published:** 2024-11-28

**Authors:** Edgar Lehr, Jiří Moravec, Yingtong Wang, Marek Uvizl

**Affiliations:** 1 Department of Biology, Illinois Wesleyan University, P.O. Box 2900, Bloomington, IL 61701, USA Illinois Wesleyan University Bloomington United States of America; 2 Departamento de Herpetología, Museo de Historia Natural, Universidad Nacional Mayor de San Marcos, Av. Arenales 1256, Jesús María, Lima 15072, Peru Universidad Nacional Mayor de San Marcos Lima Peru; 3 Department of Zoology, National Museum, Cirkusová 1740, 193 00 Prague 9, Czech Republic National Museum Prague Czech Republic; 4 Department of Zoology, Faculty of Science, Charles University, Viničná 7, Praha 2, Czech Republic Charles University Praha Czech Republic

**Keywords:** Andes, cryptic species diversity, Enrique Stanko Vráz, *Pristimantisvrazi* new species

## Abstract

Herpetological inventories conducted in the Pui Pui Protected Forest in the central Peruvian Andes between 2012 and 2014 revealed unusually high local anuran richness and endemism. Herein, we describe a new species of *Pristimantis* discovered in the buffer zone of the protected area between 1550 and 1730 m a.s.l. The description is based on one subadult male (snout–vent length 14.4 mm), one adult female (snout–vent length 26.4 mm), and six juvenile specimens collected in the montane forest between 1550 and 1730 m a.s.l. DNA barcoding placed *P.vrazi***sp. nov.** as the sister taxon to *P.rhabdocnemus* and in the clade also containing *P.lindae*, *P.sinschi*, *P.quaquaversus*, and one still unnamed *Pristimantis* species. *Pristimantisvrazi***sp. nov.** differs from all these closely related species by the combination of the following characters: tuberculate dorsum, presence of the tympanum, presence of dentigerous processes on the vomer, absence of vocal slits, a red median horizontal streak across the iris, a narrow black median vertical streak on the lower half of the eye, cream to dark brown dorsal ground coloration, and cream to gray ventral ground coloration.

## ﻿Introduction

**Figure 1.**
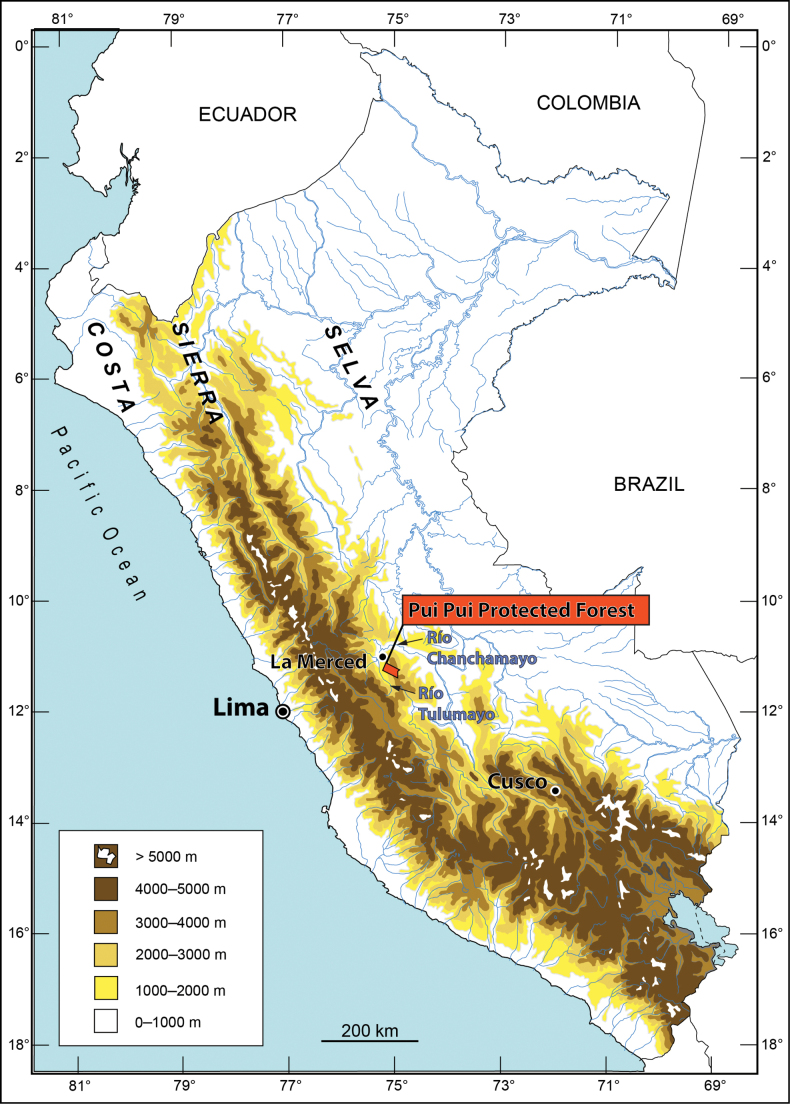
Map of Peru with the Pui Pui Protected Forest (Junín Region) in red.

**Figure 2.**
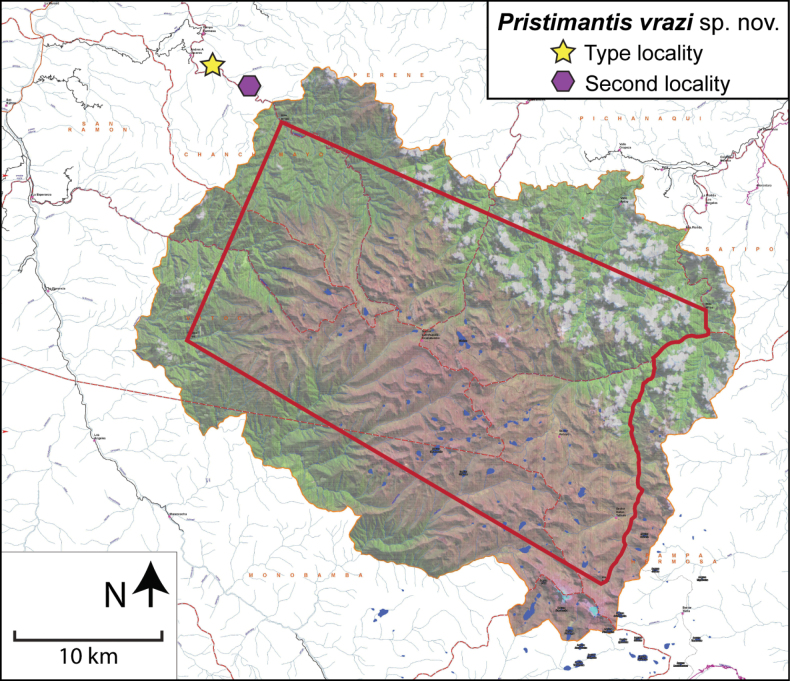
Map of the Pui Pui Protected Forest with the collecting sites of *Pristimantisvrazi* sp. nov.

The Pui Pui Protected Forest (PPPF, Figs 1, 2), located in eastern Andes of central Peru, was established in 1985 and covers 60,000 ha (45.5% montane forest, 54.5% puna habitats) between 1700 and 4500 m a.s.l. Its herpetofauna was unknown until Lehr and colleagues started surveys between 2012 and 2014 in both upper montane forest and high Andean grasslands (puna). Their expeditions resulted in the recording of 37 species for the PPPF (22 amphibians, 15 reptiles; Lehr and Moravec 2024), including seven new species of amphibians (six species of the genus *Pristimantis* Jiménez de la Espada, 1870, and one *Phrynopus* Peters, 1873), one new species of gymnophthalmid lizard, and two new gymnophthalmid genera (Lehr and Moravec 2017; Lehr and von May 2017; Lehr et al. 2017b, 2017c, 2018, 2020; Moravec et al. 2018, 2020).

A recent DNA barcoding of an undetermined series of *Pristimantis* obtained in 2013 from montane forests of the PPPF buffer zone (*Pristimantis* sp. 1 and *P.* sp. 2 of Lehr and Moravec 2024) revealed the presence of three species: one clustering together with *P.croceoinguinis* (Lynch, 1968), and two unnamed species that are new to science.

Because one of the new species is only presented by a single barcoded specimen, we hesitate to describe it as a new until additional molecular data support its species status. The second new species is represented by a series of eight specimens and distinguished based on two barcoded adult individuals. Therefore, we describe it herein as a new species of *Pristimantis*, which phenotypically resembles *P.croceoinguinis*, *P.lindae* (Duellman, 1978), *P.rhabdocnemus* (Duellman & Hedges, 2005), and *P.sinschi* Lehr, Moravec & Kodejš, 2020.

## ﻿Material and methods

### ﻿Morphological characters

The format for the description follows Lynch and Duellman (1997), except that the term “dentigerous processes of vomers” is used instead of “vomerine odontophores” (Duellman et al. 2006), and the diagnostic characters used are those of Duellman and Lehr (2009). Taxonomic classification follows Hedges et al. (2008), except that we followed Pyron and Wiens (2011) for family placement. The holotype was fixed in 96% ethanol and stored in 70% ethanol. Liver tissue of the holotype and paratype were taken for genetic analyses. Sex and maturity of specimens were identified by observing gonads by dissections. Specimens were considered juveniles when gonads were too small to distinguish between sexes. A specimen with clearly identifiable gonads but with only a slightly larger snout-vent length compared to the largest juvenile was considered a subadult. The following measurements were taken by YW to the nearest 0.1 mm with digital calipers under a stereomicroscope: snout–vent length (**SVL**, straight length distance from tip of snout to vent), tibia length (**TL**, distance from the knee to the distal end of the tibia), foot length (**FL**, distance from proximal margin of inner metatarsal tubercle to tip of toe IV), head length (**HL**, from angle of jaw to tip of snout), head width (**HW**, at level of angle of jaw), horizontal eye diameter (**ED**), interorbital distance (**IOD**), upper eyelid width (**EW**), internarial distance (**IND**), eye–nostril distance (**E–N**, straight line distance between anterior corner of orbit and posterior margin of external nares). Fingers and toes are numbered preaxially to postaxially from I–IV and I–V, respectively. We compared the lengths of toes III and V by adpressing both toes against toe IV; lengths of fingers I and II were compared by adpressing the fingers against each other. Drawings were made by YW using a stereomicroscope and a camera lucida. Photographs taken by EL and JM were used for descriptions of coloration in life. Coloration refers to coloration in life unless otherwise stated. Holotype was photographed by YW submersed in ethanol to avoid reflections. Comparisons of congeners focused on phenotypically similar species from Ecuador and Peru, those with close phylogenetic relationships as recovered in our tree, and species known from the PPPF and its buffer zone. Information on species for comparative diagnoses was obtained from Duellman and Lehr (2009), from original species descriptions, and from examined specimens. For specimens examined, see Appendix 1 “Comparative specimens examined”. Codes of collections are: **IWU** (field number) = Illinois Wesleyan University, Bloomington, USA; **MUSM** = Museo de Historia Natural Universidad Nacional Mayor de San Marcos, Lima, Peru; **NMP-P6V** = National Museum Prague, Prague, Czech Republic. Threat status was assessed using the IUCN criteria (IUCN 2016).

### ﻿Molecular analysis

#### ﻿Taxon sampling

In this study, we used liver samples of three *Pristimantis* species collected by us in the buffer zone of the PPPF in 2013 to extract DNA. A list of the newly genetically investigated material and its GenBank accession numbers is in Table 1. For the final dataset, we retrieved sequences of numerous additional species of *Pristimantis* from the central Peru stored in the GenBank to show phylogenetic position of our new material in relation to DNA sequences published earlier (most importantly in Duellman and Hedges 2007; Hedges et al. 2008; Lehr and Moravec 2017; Lehr and von May 2017; Lehr et al. 2017b; Moravec et al. 2020). To obtain more general phylogeny, we also selected and included into our dataset sequences of species representing all main evolutionary lineages and species groups of *Pristimantis* (Padial et al. 2014). As an outgroup, we included GenBank sequences of *Phrynopus*, *Oreobates* Jiménez de la Espada, 1872, *Tachiramantis* Heinicke, Barrio-Amorós & Hedges, 2015, and *Yunganastes* Padial, Castroviejo-Fisher, Köhler, Domic & De la Riva, 2007.

**Table 1.** Names of taxa, museum numbers, field data, and GenBank accession numbers of the newly genetically investigated material. PPPF = Pui Pui Protected Forest.

Species	Museum number	Locality	Coordinates	Elevation (m)	Collectors; year	GenBank accession number	
						*16S*	*12S*
*P.vrazi* sp. nov. (holotype)	MUSM 41581	PPPF buffer zone, Peru	11°05'44.2"S, 75°13'39.8"W	1550	E. Lehr, J. C. Cusi, R. von May, J. Moravec; 2013	PQ330255	PQ345842
*P.vrazi* sp. nov.	MUSM 41582	PPPF buffer zone, Peru	11°05'44.2"S, 75°13'39.8"W	1550	E. Lehr, J. C. Cusi, R. von May, J. Moravec; 2013	PQ330256	PQ345843
*P.croceoinguinis*	MUSM 31930	PPPF buffer zone, Peru	11°05'44.2"S, 75°13'39.8"W	1550	E. Lehr, J. C. Cusi, R. von May, J. Moravec; 2013	PQ330254	PQ345841
*Pristimantis* sp.	MUSM 32735	PPPF buffer zone, Peru	11°12'38.5"S, 74°57'28.9"W	1800	E. Lehr, J. Moravec; 2014	PQ330257	PQ345844

### ﻿DNA extraction, PCR, sequencing, and sequence alignment

The genomic DNA was extracted from the tissues stored in 96% ethanol using Geneaid Genomic DNA Mini Kit. Two mitochondrial markers, fragments of the genes for 12S rRNA (*12S*) and 16S rRNA (*16S*), that are commonly used in the amphibian DNA barcoding (Vences et al. 2012), were targeted. The primer sequences and PCR conditions were adapted after previous studies (Kocher et al. 1989; Palumbi et al. 1991; Moravec et al. 2009, 2020). The PCR products were subjected to Sanger sequencing forward and reverse directions at Macrogen, Inc. (Amsterdam, the Netherlands), using the PCR primers. The multiple sequence alignment was performed using the implemented MAFFT plugin (Katoh and Standley 2013) in Geneious v. 11.0.5 (https://www.geneious.com), that was subsequently manually edited. The final concatenated alignment was 1850 bp long, consisting of 860 bp from *12S* and 990 bp from *16S*. The aligned matrix was uploaded to Zenodo (https://doi.org/10.5281/zenodo.13934869).

### ﻿Phylogenetic analysis

The phylogenetic tree was constructed using maximum likelihood (ML). The nucleotide substitution model for each partition, GTR+F+I+R7 for *12S* and TIM2+F+R7 for *16S*, was selected based on the Bayesian information criterion using ModelFinder (Kalyaanamoorthy et al. 2017). The ML analysis was done in IQ-TREE (Chernomor et al. 2016; Nguyen et al. 2015). The search for the best-scoring ML was performed by ultrafast bootstrap (UFBoot; Hoang et al. 2018) with 1000 bootstrap and 1000 topology replicates. The ML analysis was run on the CIPRES Science Gateway (Miller et al. 2010). Uncorrected *p*-distances between haplotypes were calculated in MEGA11 (Tamura et al. 2021).

## ﻿Results

### ﻿Molecular phylogenetic analyses

In this study, we generated four new sequences of both *12S* and *16S* for three species of the genus *Pristimantis* from central Peru. Combined with the GenBank sequences, the final dataset comprised 2,394 unique concatenated sequences (1,057 of *12S*, and 2,253 of *16S*) for ingroup *Pristimantis* genus and outgroup *Oreobates*, *Phrynopus*, *Tachiramantis*, and *Yunganastes*.

The final dataset was comprised of 1,306 parsimony informative positions (70.59% of the total length) and missing data accounted for 29.09% bases of the dataset.

**Figure 3.**
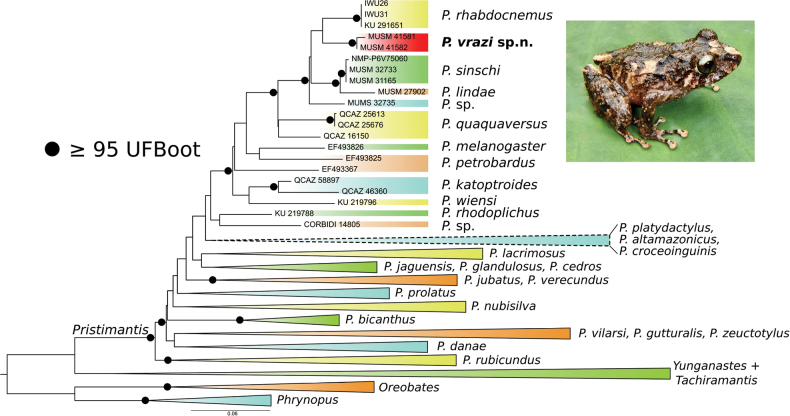
Maximum-likelihood tree of reconstructed phylogenetic relationships of the *Pristimantis* frogs based on the concatenated dataset *12S* and *16S* sequences. Branch support values are shown by black dots on the nodes. The numbers at the tips represent either voucher numbers or GenBank accession numbers. Branch support values are shown by black dots on the nodes. The clade outlined with a dotted line includes the fourth sample molecularly processed in this study, along with other species such as *P.croceoinguinis*, *P.altamazonicus*, *P.ashaninka*, *P.cruciocularis*, and *P.platydactylus*.

The obtained phylogenetic tree (Fig. 3) shows up to 11 clades within the highly supported genus *Pristimantis*. In general, the structure is consistent with clades/species groups reconstructed in our previous studies based on less numerous datasets (Lehr et al. 2017a; Moravec et al. 2020). The newly recognized species, *Pristimantisvrazi* sp. nov., belongs to a well-supported subclade with *P.lindae*, *P.quaquaversus* (Lynch, 1974), *P.rhabdocnemus*, *P.sinschi*, and *Pristimantis* sp. as closest relatives (*P.rhabdocnemus* in sister position). The mean uncorrected genetic *p*-distances values of 16S rRNA barcode between the new species and other members of this subclade vary between 5.01% and 7.52% (Table 2).

**Table 2.** Mean uncorrected genetic *p*-distance values of 16S rRNA barcode for species from the clade containing *Pristimantisvrazi* sp. nov. and for *P.croceoinguinis*. The distances are shown as percentages.

Species		1	2	3	4	5	6	7	8	9	10	11
**1**	*P.rhabdocnemus*	0.00										
**2**	*P.vrazi* sp. nov.	5.01–5.20	0.46									
**3**	*P.sinschi*	5.02–5.33	6.34–7.08	0.00								
**4**	*P.lindae*	6.80–7.22	7.08–7.52	3.05–3.06	–							
**5**	*Pristimantis* sp.	5.93–6.11	5.61–6.18	4.82	6.11	–						
**6**	*P.quaquaversus*	8.38–9.64	8.79–12.30	8.18–9.11	9.18–10.36	6.91–9.18	0.00–1.90					
**7**	*P.melanogaster*	10.05–10.69	10.65–11.21	8.81–8.86	9.16	10.22	7.40–8.88	–				
**8**	*P.petrobardus*	10.97–11.68	10.53–10.66	8.68–9.74	10.59	10.60	8.20–10.20	7.64	–			
**9**	*P.katoproides*	8.57–12.74	5.23–11.85	5.71–11.17	5.71–10.55	6.86–11.30	7.48–12.43	4.00–9.87	7.43–10.04	4.00		
**10**	*P.wiensi*	9.66–10.27	11.55–12.30	8.96–9.01	9.48	11.11	8.18–10.73	8.08	8.92	5.71–9.66	–	
**11**	*P.croceoinguinis*	16.13–16.95	16.98–17.81	13.46–13.54	14.70	14.84	12.00–14.34	11.51	11.17	12.00–15.41	13.08	–

The previous undetermined PPPF*Pristimantis* sp. 1 (sensu Lehr and Moravec 2024) clusters together with *P.croceoinguinis* and belongs to a different species-rich clade containing, for example, *P.altamazonicus* (Barbour & Dunn, 1921), *P.ashaninka* Lehr & Moravec, 2017, *P.cruciocularis* (Lehr, Lundberg, Aguilar & von May, 2006), *P.platydactylus* (Boulenger, 1903), and many other species (Fig. 3; Lehr et al. 2017a; Moravec et al. 2020).

### ﻿Taxonomy

#### ﻿ Pristimantis vrazi

Taxon classificationAnimaliaAnuraStrabomantidae

Moravec, Lehr, Wang & Uvizl sp. nov.

EC740775-4D24-5B23-9874-C22D9E9CFA5C

https://zoobank.org/BF790673-89B1-45ED-A8B6-51660A874404

**Figure 4.**
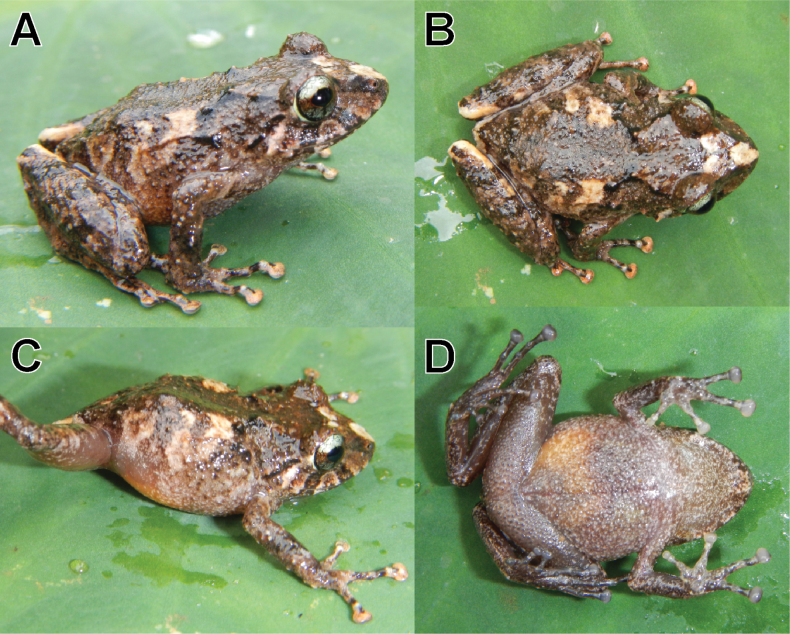
Life adult female holotype (MUSM 41581, SVL = 26.4 mm) of *Pristimantisvrazi* sp. nov. **A** dorsolateral view **B** dorsal view **C** lateral view **D** ventral view. Photos by E. Lehr.

**Figure 5.**
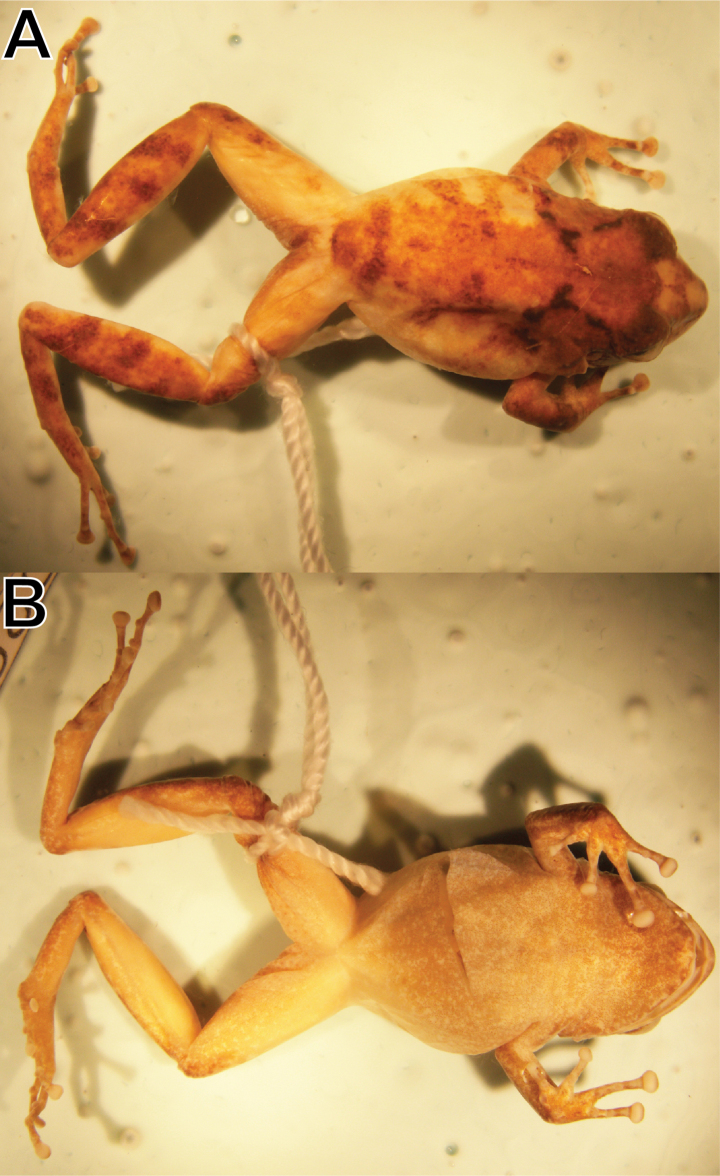
Preserved holotype (MUSM 41581, SVL = 26.4 mm) of *Pristimantisvrazi* sp. nov. **A** dorsal view **B** ventral view. Photos by Y. Wang.

**Figure 6.**
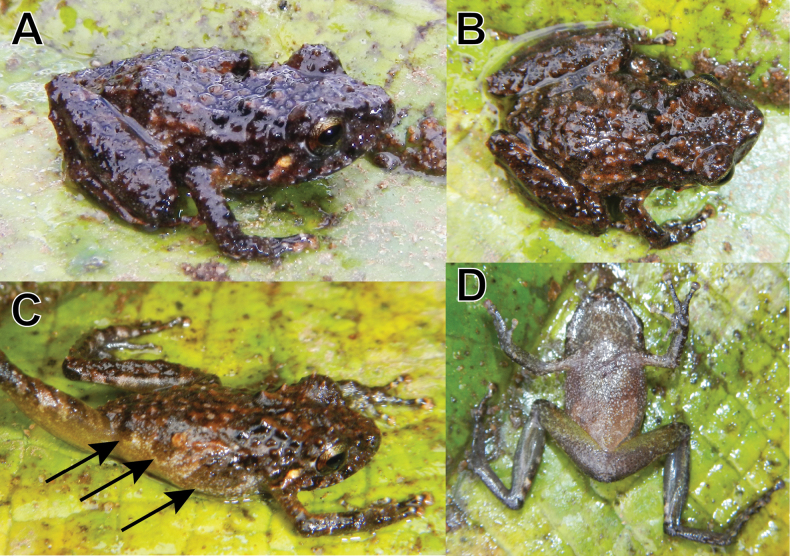
Life subadult male paratype (MUSM 48582, SVL = 14.4 mm) of *Pristimantisvrazi* sp. nov. **A** dorsolateral view **B** dorsal view **C** lateral view **D** ventral views. Arrows indicate the three dark grayish-brown bars on the flank. Photos by E. Lehr.

**Figure 7.**
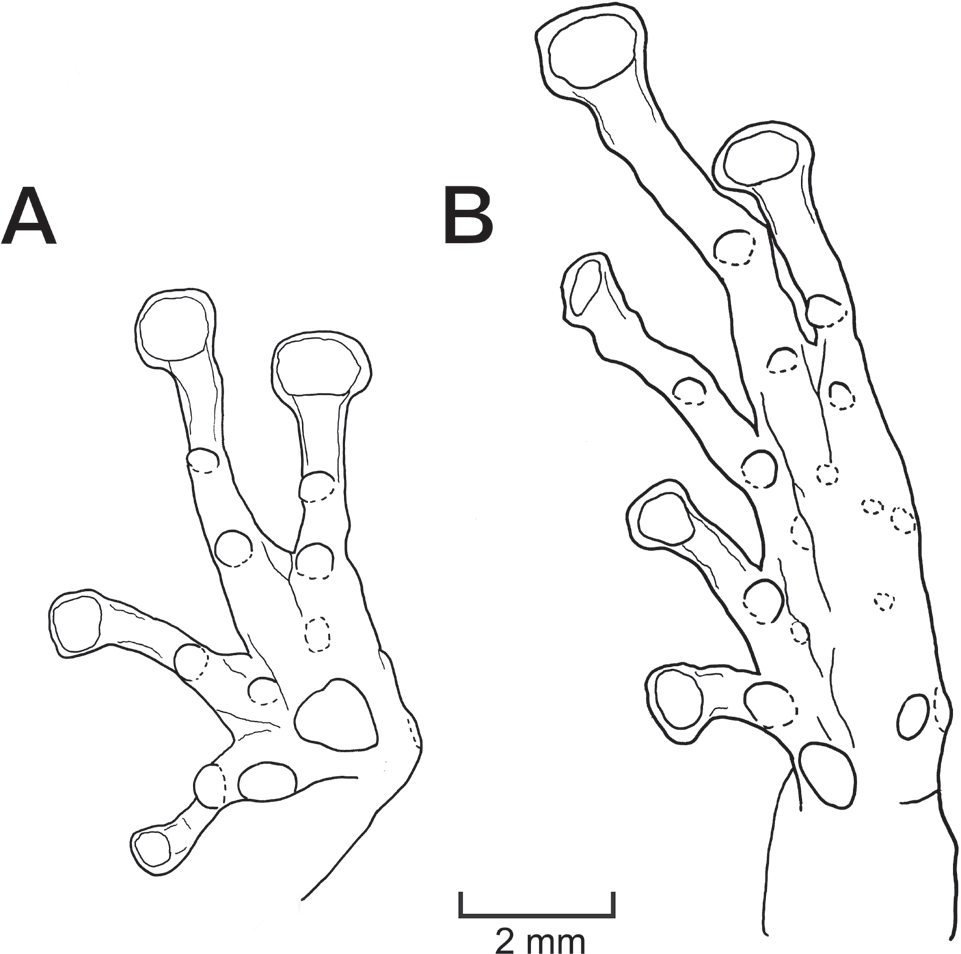
Holotype (MUSM 41581) of *Pristimantisvrazi* sp. nov. **A** ventral view of hand **B** ventral view of foot, preserved toes are not on the same plane, resulting in an artifact with toe V appearing thinner than it is. Drawings by Y. Wang.

**Figure 8.**
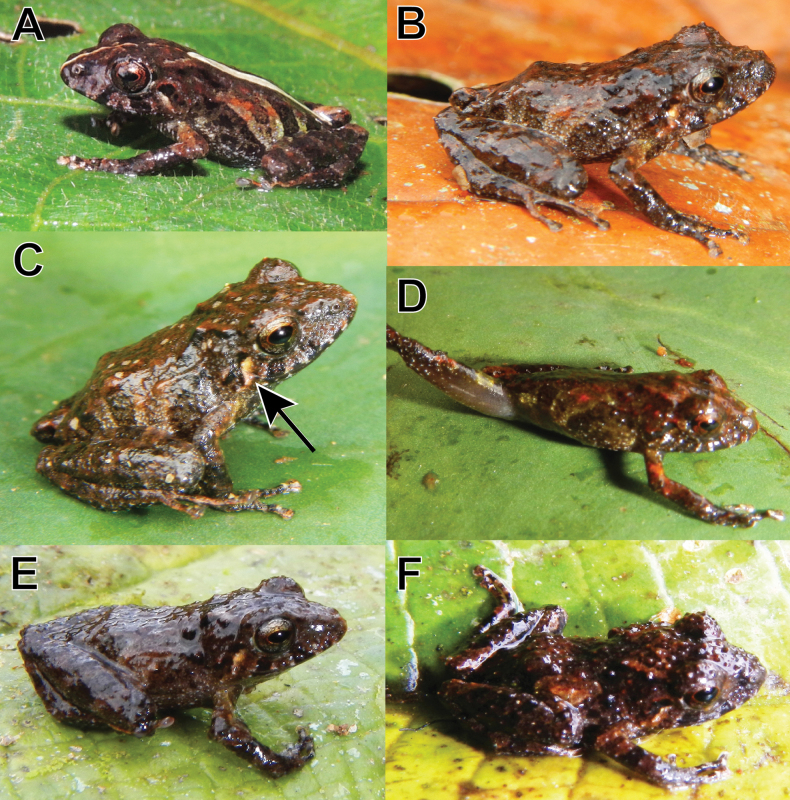
Juvenile referred specimens of *Pristimantisvrazi* sp. nov. **A**MUSM 31929, SVL = 12.3 mm **B**MUSM 31933, SVL = 12.0 mm **C**MUSM 41583, SVL = 14.1 mm **D**MUSM 31937, SVL = 11.9 mm **E**MUSM 31932, SVL = 14.3 mm **F**MUSM 31938, SVL = 12.1 mm. Photo A by J. Moravec, all others by E. Lehr.

Figs 4 , 5 , 6 , 7 , 8 , Tables 1 , 2

##### Type material.

***Holotype*.**MUSM 41581 (field number IWU 200, Figs 4, 5, 7), GenBank accession numbers PQ330255 (*16S*), PQ345842 (*12S*); adult ♀; from buffer zone of the Pui Pui Protected Forest (11°05'44.2"S, 75°13'39.8"W), 1550 m a.s.l., property of Abelardo Cabrejos Vega, Distrito Pichanaqui, Provincia Chanchamayo, Región Junín, Peru; Edgar Lehr, Juan Carlos Cusi, Rudolf von May & Jiří Moravec leg., 10 June 2013.

***Paratype*.**MUSM 41582 (IWU 205, Fig. 6), GenBank accession numbers PQ330256 (*16S*), PQ345843 (*12S*); 1 subadult ♂; from the type locality; Edgar Lehr, Juan Carlos Cusi, Rudolf von May & Jiří Moravec leg., 10 June 2013.

##### Referred specimens.

MUSM 31929 (IWU 197), MUSM 41583 (IWU 201), MUSM 31932 (IWU 204), MUSM 31933 (IWU 206) (Fig. 8), 4 juveniles; all from the type locality; Edgar Lehr, Juan Carlos Cusi, Rudolf von May, and Jiří Moravec leg.; 10 June 2013 • MUSM 31937 (IWU 214), MUSM 31983 (IWU 215), 2 juveniles; from the buffer zone of the Pui Pui Protected Forest (11°06'17.0"S, 75°12'26.2"W); 1730 m a.s.l.; Edgar Lehr, Juan Carlos Cusi, Rudolf von May, and Jiří Moravec leg.; 11 June 2013.

##### Generic placement.

We assign this species to *Pristimantis* based on our molecular data (Fig. 3) and the general morphological similarity to other members of the genus.

##### Diagnosis.

A new species of *Pristimantis* not assigned to any species group having the following combination of characters: (1) skin on dorsum shagreen with scattered conical tubercles; skin on venter areolate; weak discoidal fold present; short dorsolateral ridges present; (2) tympanic membrane and tympanic annulus present; (3) snout moderately long, acutely rounded in dorsal view, rounded in profile; (4) upper eyelid bearing small conical tubercles; EW narrower than IOD; cranial crest absent; (5) dentigerous processes of vomers present, oblique; (6) vocal slits and nuptial pads absent; (7) finger I shorter than finger II; discs of digits broadly expanded, round, bearing circumferential grooves; (8) fingers with narrow lateral fringes; (9) ulnar tubercles small, round; (10) heel without tubercles; inner tarsal fold absent; inner edge of tarsus with low, elongated tubercles; (11) inner metatarsal tubercle ovoid, 4–5 times larger than outer elongated metatarsal tubercle; numerous supernumerary plantar tubercles; (12) toes with weak lateral fringes; basal toe webbing present; toe V longer than toe III; toe discs smaller than those on fingers, circumferential grooves present; (13) in life, the dorsum ranges from cream to dark grayish brown with a black W-shaped scapular fold, with or without cream blotches; anterior and posterior surfaces of thighs grayish brown with pale gray spots; groin grayish brown with pale-gray spots with or without a hint of olive-green; venter cream to gray with or without numerous small, brown, dark dots; iris pale bronze with fine black reticulation, a reddish brown median horizontal streak, and a narrow black median vertical streak on lower half of eye; head light to dark brown dorsally, with or without brown or black interorbital bar and canthal stripes, with or without white spot at dorsal tip of snout; (14) SVL in single subadult male 14.4 mm, in single adult female 26.4 mm.

##### Comparison.

*Pristimantisvrazi* is distinguished from its congeners in Peru (152 species; AmphibiaWeb 2024) by the following combination of characters: dorsum with conical tubercles and short dorsolateral ridges, tympanic membrane and tympanic annulus present, males without vocal slits and nuptial pads, dentigerous processes of vomer present, groin grayish brown with or without a hint of pale olive-green, and iris pale bronze with fine black reticulation, a reddish-brown median horizontal streak, and a narrow black median vertical streak on lower half of eye. The following 11 species of *Pristimantis* have been recorded from montane forests in the PPPF and its surroundings including the Distrito de Pampa Hermosa (Moravec et al. 2020; Venegas et al. 2023; Lehr and Moravec 2024; this paper): *Pristimantisalbertus* Duellman & Hedges, 2007, *P.aniptopalmatus* (Duellman & Hedges, 2005), *P.ashaninka*, *P.bipunctatus* (Duellman & Hedges, 2005), *P.clarae* Venegas, García-Ayachi, Marchelie, Ormeño & Catenazzi, 2023, *P.croceoinguinis*, *P.cruciocularis*, *P.sagittulus* (Lehr, Aguilar & Duellman, 2004), *P.sinschi*, P.cf.stictogaster (Duellman & Hedges, 2005), and *P.* sp.

Comparing to *Pristimantisvrazi* (characters in parentheses), *P.albertus* has distinct dorsolateral folds (short ridges), males with vocal slits (absent), and a yellow groin (grayish brown with pale-gray spots with or without a hint of olive-green) (Duellman and Hedges 2007; Moravec et al. 2020). *Pristimantisaniptopalmatus* has females with SVL up to 22.0 mm (26.4 mm), males have vocal slits (absent), and the iris is grayish white (pale bronze with reddish-brown streak) (Duellman and Hedges 2005). *Pristimantisashaninka* is only known from the northeastern corner of the PPPF (northwestern corner), and both species have the dorsum with conical tubercles and the venter pale gray and mottled grayish brown. However, *P.ashaninka* lacks a tympanum (present), has the dorsum with a reddish-brown blotch in shape of an hourglass (absent), and the iris with a black narrow upper and lower streak (only lower streak present) (Lehr and Moravec 2017). *Pristimantisvrazi* and *P.bipunctatus* have the iris bronze, with a median horizontal reddish-brown streak. However, *P.bipunctatus* has females with SVL up to 41.5 mm (26.4 mm), males with vocal slits and nuptial pads (absent), and the scapular with a pair of black warts (absent) (Duellman and Lehr 2009). *Pristimantisclarae* was recently described from the Distrito de Pampa Hermosa, but it is a member of the *P.danae* group and differs in having distinct dorsolateral folds (short ridges), heals with low conical tubercles (absent), males with vocal slits (absent), coppery iris (pale bronze with reddish-brown streak), and dorsum with triangular blotches (dorsum dark grayish brown) (Venegas et al. 2023). *Pristimantiscroceoinguinis* is known from montane forests in the north-central buffer zone and the eastern corner of the PPPF, and it also has the iris with a reddish-brown median streak and a vertical dark-brown streak at lower half of the eye. However, *P.croceoinguinis* lacks a tympanum (present), and has the groin yellow (grayish brown) (Lynch 1968). *Pristimantisvrazi* and *P.cruciocularis* occur syntopically in the surroundings of the PPPF. However, *P.cruciocularis* lacks a tympanum (present), fingers and toes lack lateral fringes (present), has a red or orange groin (cream to dark brown with or without a hint of olive-green), and has the iris with a dark-brown cross (vertical streak at lower half or iris) (Lehr et al. 2006).

*Pristimantissagittulus* from the northwestern part of the PPPF has dentigerous processes of vomers absent (present), males with vocal slits and nuptial pads (both absent), and posterior surfaces of thighs with longitudinal red stripes (no red skin coloration) (Duellman and Lehr 2009). *Pristimantisstictogaster* has vocal slits present (absent), and dark-brown groin with white spots (grayish brown with pale-gray spots with or without a hint of olive-green), and white belly with dark-brown spots (venter cream to gray with or without numerous small dark-brown dots) (Duellman and Lehr 2009). *Pristimantisplatydactylus* is known for its remarkable variation in color pattern (De la Riva 1998; Lehr et al. 2006; Duellman and Lehr 2009). However, *P.platydactylus* has the tympanic annulus barely evident (distinct), and males have vocal slits and nuptial pads (both absent).

Our phylogeny (Fig. 3) revealed close phylogenetic similarities of *P.vrazi* with *P.lindae*, *P.quaquaversus*, *P.rhabdocnemus*, *P.sinschi*, and *P.* sp. *Pristimantisvrazi* and *P.lindae* have the iris with a red median horizontal streak and fingers and toes with lateral fringes. However, *P.lindae* has the dorsum shagreen (tuberculate), males with nuptial pads and vocal slits (absent), and a prominent tympanum (not prominent) (Duellman 1978). *Pristimantisquaquaversus* has males with vocal slits (absent), heel with a conical tubercle (absent), tympanic membrane absent (present), and the venter white with or without brown spots (gray and brown mottled) (Lynch 1974). *Pristimantisvrazi* and *P.rhabdocnemus* are related as sister taxa in our phylogeny (Fig. 3), are of similar size, and have a grayish-brown dorsal ground coloration. However, *P.rhabdocnemus* lacks a tympanum (present), lacks dentigerous processes of vomers (present), and has a grayish-tan iris (iris pale bronze with fine black reticulation, a reddish-brown median horizontal streak, and a narrow black median vertical streak on lower half of eye) (Duellman and Hedges 2005). *Pristimantisvrazi* and *P.sinschi* have the dorsum with conical tubercles, the venter areolate, and males that lack vocal slits and nuptial pads, and the iris pale bronze with fine black reticulation and broad median red or reddish-brown band through pupil and a narrow black vertical streak from pupil across lower half of iris (Moravec et al. 2020; this paper). However, *P.sinschi* lacks a tympanum (present), lacks a pale brown tympanic stripe (present), has the groin black with cream blotches (cream to dark brown with or without a hint of olive-green), and venter mottled black and cream (pale gray and brown mottled).

##### Holotype description.

Adult female (Figs 4, 5); head narrower than body, slightly longer than wide; head width 36.4% of SVL; head length 33.3% of SVL; cranial crest absent; snout acutely rounded in dorsal view, round and moderate in length in lateral view, E–N 78.3% of eye diameter; nostrils protuberant, directed dorsolaterally; canthus rostralis straight in dorsal view, rounded in profile; loreal region slightly concave; lips rounded; upper eyelid bearing several small, conical tubercles; EW 85.2% of IOD; supratympanic fold distinct, short, extending diagonally from posterior margin of upper eyelid towards insertion of arm, covering upper and posterior margin of the tympanum; tympanic membrane and tympanic annulus present, more distinct on the right side of head, tympanum 29.3% of ED; several conical postrictal tubercles present bilaterally, some fusing to short ridges. Choanae small, ovoid; dentigerous processes of vomers oblique and round; tongue longer than wide, not notched posteriorly, posterior one-third free.

Skin on dorsum (Fig. 4B) shagreen, with many scattered tubercle, with short, weakly defined dorsolateral fold on anterior half of body (Fig. 4A); skin on flanks shagreen with scattered tubercles (Fig. 4C); skin on throat and chest smooth, belly areolate (Fig. 4D); discoidal and thoracic folds weakly defined; cloacal sheath short.

Outer ulnar surface with numerous minute tubercles; outer palmar tubercle round and slightly bifid, inner palmar tubercle ovoid, approximately one-half size of outer tubercle; distinct supernumerary tubercles, ovoid, approximately one-half size of subarticular tubercles; subarticular tubercles well defined, round in ventral view, conical in lateral view; fingers with narrow lateral fringes; finger I shorter than finger II; discs on fingers broadly expanded, rounded, bearing circumferential grooves (Fig. 7A).

Hind limbs long, slender, tibia length 53.1% of SVL; foot length 43.6% of SVL; upper surface of hind limbs shagreen with many scattered tubercles; anterior surface of thighs smooth, posterior and ventral surfaces of the thighs areolate; heels without conical tubercles; outer surface of tarsus with minute tubercles; inner tarsal fold not present; inner metatarsal tubercle ovoid, 4–5 times size of ovoid outer metatarsal tubercle; subarticular tubercles well defined, round in ventral view, conical in lateral view; few plantar supernumerary tubercles distinct, about one-fourth size of subarticular tubercles; toes with narrow lateral fringes; basal webbing present, most prominent between toes IV and V; discs expanded, slightly truncated, slight smaller as discs on fingers, bearing circumferential grooves; relative length of toes: I <II<III<V<IV (Fig. 7B).

In life (Fig. 4), dorsum dark grayish brown with a black W-shaped scapular fold; head dorsally with a pale brown interorbital bar and a pale cream blotch on snout, head laterally grayish brown with a dark-brown canthal stripe, a dark-brown supratympanic stripe, two dark-brown labial bars below eye and distinct pale brown tympanic stripe from posterior outer corner of upper eyelid to corner of mouth; arms dorsally grayish brown with a dark-brown diagonal bar on lower arm; hind limbs dorsally grayish brown with three dark-brown diagonal bars; flanks pale brown with three diagonal dark grayish-brown bars alternating with three pale-brown diagonal to vertical bars; groin, anterior and posterior surfaces of thighs grayish brown with pale-gray spots; throat, chest, belly, and extremities pale-gray and dark-brown mottled; finger and toe discs dorsally pale reddish brown; iris pale bronze with fine black reticulation, a reddish-brown median horizontal streak, and a narrow black median vertical streak at lower half of eye.

In alcohol (Fig. 5) after 10 years, the dorsal ground coloration is pale brown with dark-brown flecks, dark brown W-shaped ridge in scapular area, cream interorbital bar and cream blotch on snout; flanks cream with three pale-brown stripes; groin, anterior and posterior surfaces of thighs cream; thighs and tibias ventrally cream, throat, chest, and belly cream and pale grayish-brown mottled; iris pale gray.

Holotype measurements (in mm). SVL 26.4; TL14.0; FL 11.5; HL 8.8; HW 9.6; ED 3.7; TY 1.1; IOD 2.7; EW 2.3; IND 2.1; E–N 2.9.

##### Variation.

The single subadult male paratype MUSM 41582 (Fig. 6) has following measurements (in mm): SVL 14.4; TL 8.2; FL 6.3; HL 5.0; HW 5.4; ED 2.3; TY 0.4; IOD 2.2; EW 1.2; IND 1.2; E–N 1.6. It has strongly tuberculated dorsum (tubercles of conical shape), dark grayish-brown dorsum with reddish-brown flecks. The flanks bear three dark grayish-brown bars (Fig. 6C). The six juvenile referred specimens (Fig. 8, SVL = 11.9–14.3 mm) have the dorsum tuberculate, the venter pale to dark gray, with brown mottling, the iris pale bronze with fine black reticulation, a reddish-brown median horizontal streak, and a narrow black median vertical streak on lower half of the eye, and a distinct pale-cream tympanic stripe (Fig. 8C). One specimen (MUSM 31929, Fig. 8A) has a middorsal pale cream stripe that is narrow on the head and wider on its back.

##### Etymology.

We dedicate this new species to the Czech explorer and patriot Enrique Stanko Vráz (1860–1932), who explored Africa, South America, and eastern Asia (Todorová 2006). In South America, he spent several years working in Venezuela and traveling by boat from Venezuela to Peru via the Rio Orinoco, Rio Negro, and Rio Amazonas before crossing the Andes and working around Cajamarca. His specimen collections (animals, artifacts) were sent to the National Museum in Prague. He published his travels and observations in South America in a book (Vráz 1900) that provides valuable original insights into South American nature and indigenous peoples at the end of the 19^th^ century and still inspires people. The specific epithet is used as a noun in apposition.

##### Distribution and natural history.

**Figure 9.**
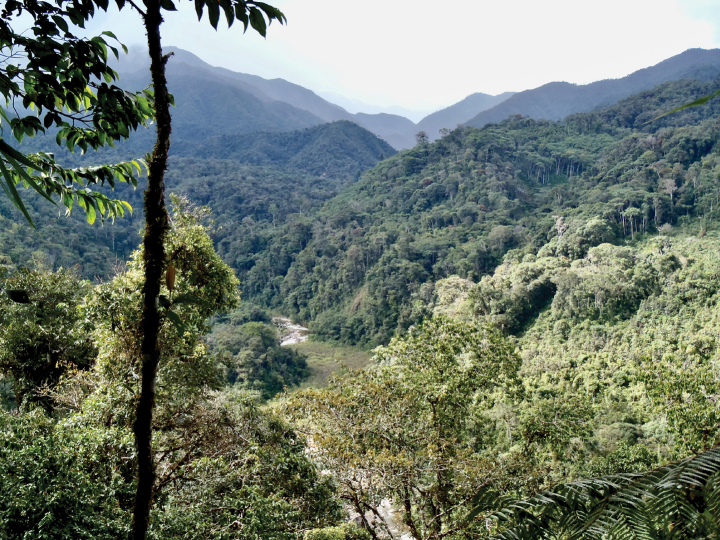
Valley of the Rio Huatziroqui in the buffer zone of the Pui Pui Protected Forest, type locality of *Pristimantisvrazi* sp. nov. Type specimens were collected along the river at elevations between 1550 m and 1730 m a.s.l. Photo by J. Moravec.

*Pristimantisvrazi* is only known from two localities of the northwestern corner of the buffer zone of the Pui Pui Protected Forest (Fig. 2) between 1550 and 1730 m a.s.l. The type locality lies in the valley of Rio Huatziroqui (Fig. 9). The slopes of the valley are covered by primary mountain rainforest characterized by a 15–20 m high canopy. The type specimens were collected on the right bank of the river. They inhabited a disturbed forest that was interspersed with small coffee plantations, solitary houses of local coffee farmers, and various forest paths. All specimens were found at night on low vegetation (up to 80 cm above ground). The holotype was found at 8:55 p.m. on a leaf 80 cm above ground. Other syntopic frogs include *Pristimantisbipunctatus*, *P.cruciocularis*, and the hylid *Boanaaguilari*. Considering the sparse data available, we here classify *P.vrazi* as Data Deficient according to the IUCN Red List criteria.

## ﻿Discussion

It is a common practice for taxonomists to describe at first the “easy” new species, those that are easily recognized based on unique characters, and leave the more challenging taxa for later. Lehr and colleagues followed this approach and described six new species from the PPPF in 2017, one new reptile species and genus in 2018, a new reptile genus in 2019, and one new species in 2020 (Lehr and von May 2017; Lehr and Moravec 2017; Lehr et al. 2017b, 2017c, 2018, 2020; Moravec et al. 2018, 2020). A series of 11 specimens of *Pristimantis* sp. that were difficult to identify were kept in a jar, and we assumed that they represented one new species. Hence, we collected tissues selectively to cover the different collecting sites. Surprisingly, our molecular phylogeny (Fig. 3) revealed three species of *Pristimantis*. One is *P.croceoinguinis* (listed as *Pristimantis* sp. 1 and shown in photos 63 and 64 in Lehr and Moravec (2024). Two other species are new to science. The first of them is named *Pristimantis* sp. 2 by Lehr and Moravec (2024; photos 65 and 66) and *Pristimantis* sp. in this paper; at present, it is represented by a single barcoded sample, and more robust molecular data support is necessary for its proper delimitation. The second new species (not pictured by Lehr and Moravec 2024) is described herein as *P.vrazi*. According to Venegas et al. (2023), 34 new *Pristimantis* species were discovered in the Cordillera Oriental in central Peru (Regións Huánuco, Pasco, and Junín) in the last two decades. Including their *P.clarae* and our *P.vrazi*, this number has increased to 36 species. Venegas et al. (2023) pointed out that many species of *Pristimantis* are endemic to small montane areas and valleys where they may be threatened by habitat destruction. Our herpetological surveys show that local endemism of the *Pristimantis* species is very high in the PPPF. Up to date, 15 *Pristimantis* species have been recognized in this protected area (including the puna habitats) and its buffer zone, and eight of them (53%) appear to be endemic. Comparison of *Pristimantis* diversity in three most thoroughly surveyed part of the PPPF—Rio Huatziroqui valley (8 species/2 endemics), Rio Bravo valley (5 species/2 endemics), and central and southeastern part of the puna zone (4 species/4 endemics)—reveals that the local endemism reaches 25–100%. This finding, which underlines the exceptionality of the local anuran fauna, can be explained by the geography of the PPPF. The protected area is located on the northwestern spur of the eastern Andes, which has the character of a mountainous peninsula. The upper part of this peninsula is covered with puna, and its western, northern, and eastern edges descend into deep valleys covered with cloud forests and mountain rainforests. Individual valleys are isolated from each other by mountain ridges. Members of the genus *Pristimantis* generally have low vagility (Duellman and Lehr 2009). They may, therefore, be a case where allopatric speciation contributed to the emergence of many local, geographically isolated species. Similarly, a high rate of local *Pristimantis* endemism can also be detected in other isolated mountain areas in central Peru. For example, data on the anuran diversity of the Cordillera Yanachaga (Pasco Region) available in Angulo et al. (2016) and Frost (2024) show a similar level of local endemism in the genus *Pristimantis*—nine (53%) of the 17 observed species are endemic. Protected areas such as the PPPF or Yanachaga-Chemillén National Park are, therefore, of fundamental importance for the protection of the unique anuran diversity of the eastern Andes of central Peru.

Dedicating new species to people (eponyms), especially in the past or from the past, has caused public protest when their misconduct, racism, or misogyny was revealed, and demands to change the popular names have arose (e.g., North American bird species; Ogden 2024). To avoid such problems, we have carefully reviewed Vráz’s biography and publications (e.g., Vráz 1900; Todorová 2006) and did not notice any misconduct or dishonorable behavior. Consequently, we feel reassured to honor Enrique Stanko Vráz for his accomplishments in the exploration of South American nature with this species’ dedication. His books, unfortunately not translated into English, still inspire many readers.

## Supplementary Material

XML Treatment for Pristimantis vrazi

## ﻿Additional information

### Author ORCIDs

Edgar Lehr https://orcid.org/0000-0002-0517-390X

Jiří Moravec https://orcid.org/0000-0003-4114-7466

Marek Uvizl https://orcid.org/0000-0001-6366-8567

### Data availability

All of the data that support the findings of this study are available in the main text or Supplementary Information.

## ﻿Appendix 1

**Comparative specimens examined.**

*Pristimantisalbertus* (5): Peru, Junín: Pui Pui Protected Forest (Rio Huatziroqui valley, 11°07'37.2"S, 75°10'37.0"W), 1970 m a.s.l., MUSM 31953, 31956, 31959, NMP-P6V 75067, 76021.

*Pristimantisaniptopalmatus* (2): Peru, Junín: buffer zone of the Pui Pui Protected Forest, 1970 m a.s.l., MUSM 33996, NMP-P6V 75055.

*Pristimantisbipunctatus* (2): Peru, Junín: buffer zone of the Pui Pui Protected Forest (11°05'44.2"S, 75°13'39.8"W), 1550 m a.s.l., MUSM 31926, 31931.

*Pristimantiscruciocularis* (2): Peru, Junín: buffer zone of the Pui Pui Protected Forest (11°06'17.0"S, 75°12'26.2"W), 1730 m a.s.l., MUSM 31935, 31939.

*Pristimantiscroceoinguinis* (3): Peru, Junín: buffer zone of the Pui Pui Protected Forest (11°05'44.2"S, 75°13'39.8"W), 1550 m a.s.l., MUSM 31930; buffer zone of the Pui Pui Protected Forest (11°06'17.0"S, 75°12'26.2"W), 1730 m a.s.l., MUSM 31958; buffer zone of the Pui Pui Protected Forest, San Juan de Miraflores (11°06'19.3"S, 75°00'02.5"W), 1400 m a.s.l., MUSM 31154.

*Pristimantislindae* (3): Peru, Cusco: Alto Shima, 1785 m a.s.l., MUSM 26528, 26542; Alto Shima, 1790 m a.s.l., MUSM 26540.

*Pristimantissagittulus* (2): Peru: Junín: from the Pui Pui Protected Forest (Rio Huatziroqui valley, 11°07'37.2"S, 75°10'37.0"W), 1970 m a.s.l., MUSM 31952.

*Pristimantissinschi* (2): Peru: Junín: from the Pui Pui Protected (Rio Bravo valley, 11°12'38.5"S, 74°57'28.9"W), 1800 m a.s.l., MUSM 32733, NMP-P6V 75060.

Pristimantiscf.stictogaster (2): Peru, Junín: from the Pui Pui Protected Forest (Rio Bravo valley, 11°12'38.5"S, 74°57'28.9"W), ca. 1800 m a.s.l., NMP-P6V 75061, 76024.

*Pristimantis* sp. (1): Peru, Junín: from the Pui Pui Protected Forest (Rio Bravo valley, 11°12'38.5"S, 74°57'28.9"W), ca. 1800 m a.s.l., MUSMS 32735.

## Supplementary materials

Supplementary material 1 Large tree figure Data type pdf Explanation note Large tree figure: Maximum-likelihood tree showing the reconstructed phylogenetic relationships of *Pristimantis* frogs based on concatenated *12S* and *16S* sequences. The numbers at the tips represent GenBank accession numbers, except for the four samples used in this study, which are represented by voucher numbers.. https://binary.pensoft.net/file/1184914This dataset is made available under the Open Database License (http://opendatacommons.org/licenses/odbl/1.0/). The Open Database License (ODbL) is a license agreement intended to allow users to freely share, modify, and use this Dataset while maintaining this same freedom for others, provided that the original source and author(s) are credited.Edgar Lehr, Jiří Moravec, Yingtong Wang, Marek Uvizl
