# Supplementary material for: ﻿A new species of Pristimantis (Amphibia, Anura, Strabomantidae) from a montane forest of the Pui Pui Protected Forest in central Peru
Source: Zookeys. 2024 Nov 28;1219:143–63. doi: 10.3897/zookeys.1219.129773 (PMC11621819; doi:10.3897/zookeys.1219.129773)
Supplement: Supplementary material 1 — Large tree figure [file zookeys-1219-143_article-129773__-s001.pdf]

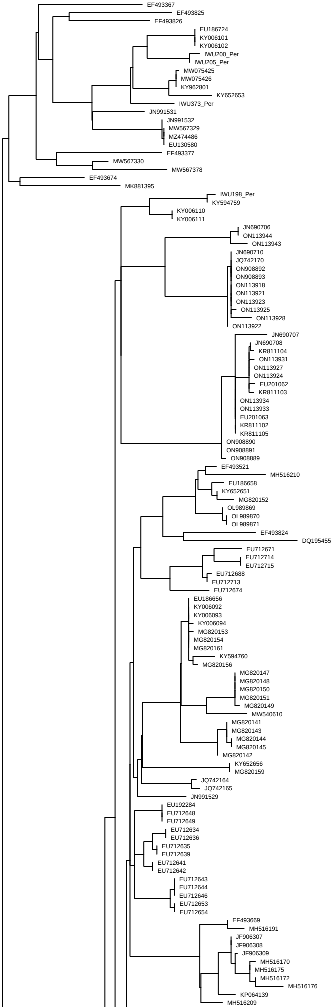
EF493367
EF493825
EF493826
EU186724
KY006101
KY006102
IWU200_Per
IWU205_Per
MW075425
MW075426
KY962801
KY652653
IWU373_Per
JN991531
JN991532
MW567329
MZ474486
EU130580
EF493377
MW567330
MW567378
EF493674
MK881395
IWU198_Per
KY594759
KY006110
KY006111
JN690706
ON113944
ON113943
JN690710
JQ742170
ON908892
ON908893
ON113918
ON113921
ON113923
ON113925
ON113928
ON113922
JN690707
JN690708
KR811104
ON113931
ON113927
ON113924
EU201062
KR811103
ON113934
ON113933
EU201063
KR811102
KR811105
ON908890
ON908891
ON908889
EF493521
MH516210
EU186658
KY652651
MG820152
OL989869
OL989870
OL989871
EF493824
DQ195455
EU712671
EU712714
EU712715
EU712688
EU712713
EU712674
EU186656
KY006092
KY006093
KY006094
MG820153
MG820154
MG820161
KY594760
MG820156
MG820147
MG820148
MG820150
MG820151
MG820149
MW540610
MG820141
MG820143
MG820144
MG820145
MG820142
KY652656
MG820159
JQ742164
JQ742165
JN991529
EU192284
EU712648
EU712649
EU712634
EU712636
EU712635
EU712639
EU712641
EU712642
EU712643
EU712644
EU712646
EU712653
EU712654
EF493669
MH516191
JF906307
JF906308
JF906309
MH516170
MH516175
MH516172
MH516176
KP064139
MH516209

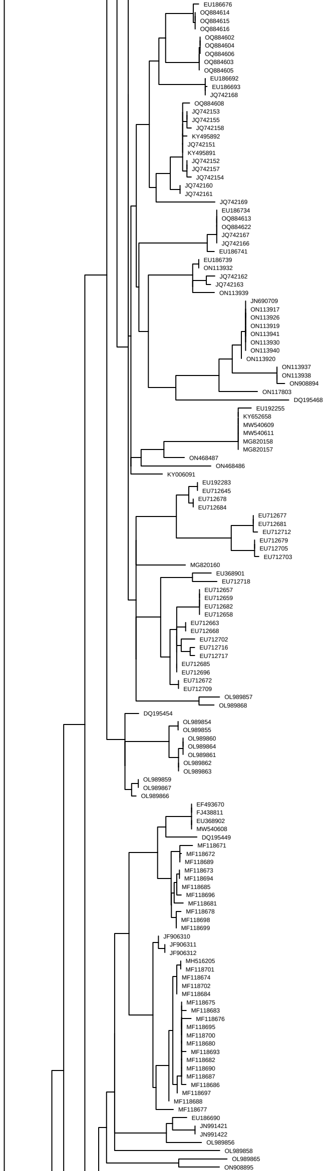
EU186676
OQ884614
OQ884615
OQ884616
OQ884602
OQ884604
OQ884606
OQ884603
OQ884605
EU186692
EU186693
JQ742168
OQ884608
JQ742153
JQ742155
JQ742158
KY495892
JQ742151
KY495891
JQ742152
JQ742157
JQ742154
JQ742160
JQ742161
JQ742169
EU186734
OQ884613
OQ884622
JQ742167
JQ742166
EU186741
EU186739
ON113932
JQ742162
JQ742163
ON113939
JN690709
ON113917
ON113926
ON113919
ON113941
ON113930
ON113940
ON113920
ON113937
ON113938
ON908894
ON117803
DQ195468
EU192255
KY652658
MW540609
MW540611
MG820158
MG820157
ON468487
ON468486
KY006091
EU192283
EU712645
EU712678
EU712684
EU712677
EU712681
EU712712
EU712679
EU712705
EU712703
MG820160
EU368901
EU712718
EU712657
EU712659
EU712682
EU712658
EU712663
EU712668
EU712702
EU712716
EU712717
EU712685
EU712696
EU712672
EU712709
OL989857
OL989868
DQ195454
OL989854
OL989855
OL989860
OL989864
OL989861
OL989862
OL989863
OL989859
OL989867
OL989866
EF493670
FJ438811
EU368902
MW540608
DQ195449
MF118671
MF118672
MF118689
MF118673
MF118694
MF118685
MF118696
MF118681
MF118678
MF118698
MF118699
JF906310
JF906311
JF906312
MH516205
MF118701
MF118674
MF118702
MF118684
MF118675
MF118683
MF118676
MF118695
MF118700
MF118680
MF118693
MF118682
MF118690
MF118687
MF118686
MF118697
MF118688
MF118677
EU186690
JN991421
JN991422
OL989856
OL989858
OL989865
ON908895

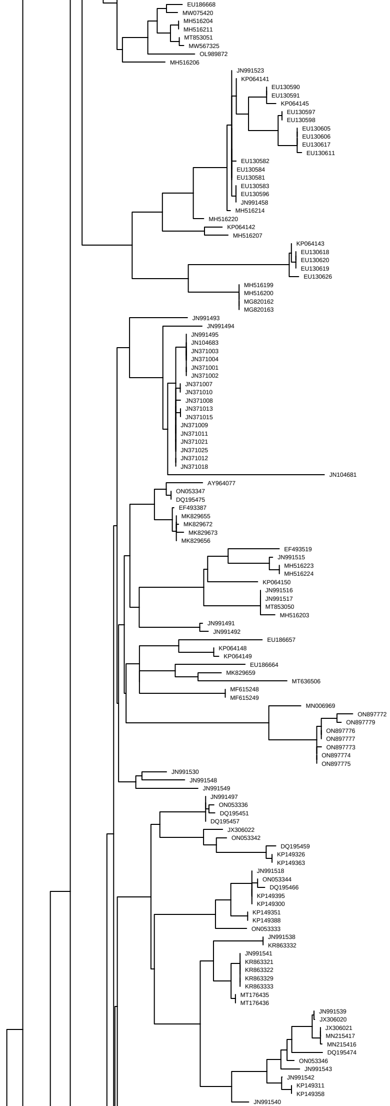
EU186668
MW075420
MH516204
MH516211
MT853051
MW567325
OL989872
MH516206
JN991523
KP064141
EU130590
EU130591
KP064145
EU130597
EU130598
EU130605
EU130606
EU130617
EU130611
EU130582
EU130584
EU130581
EU130583
EU130596
JN991458
MH516214
MH516220
KP064142
MH516207
KP064143
EU130618
EU130620
EU130619
EU130626
MH516199
MH516200
MG820162
MG820163
JN991493
JN991494
JN991495
JN104683
JN371003
JN371004
JN371001
JN371002
JN371007
JN371010
JN371008
JN371013
JN371015
JN371009
JN371011
JN371021
JN371025
JN371012
JN371018
JN104681
AY964077
ON053347
DQ195475
EF493387
MK829655
MK829672
MK829673
MK829656
EF493519
JN991515
MH516223
MH516224
KP064150
JN991516
JN991517
MT853050
MH516203
JN991491
JN991492
EU186657
KP064148
KP064149
EU186664
MK829659
MT636506
MF615248
MF615249
MN006969
ON897772
ON897779
ON897776
ON897777
ON897773
ON897774
ON897775
JN991530
JN991548
JN991549
JN991497
ON053336
DQ195451
DQ195457
JX306022
ON053342
DQ195459
KP149326
KP149363
JN991518
ON053344
DQ195466
KP149395
KP149300
KP149351
KP149388
ON053333
JN991538
KR863332
JN991541
KR863321
KR863322
KR863329
KR863333
MT176435
MT176436
JN991539
JX306020
JX306021
MN215417
MN215416
DQ195474
ON053346
JN991543
JN991542
KP149311
KP149358
JN991540

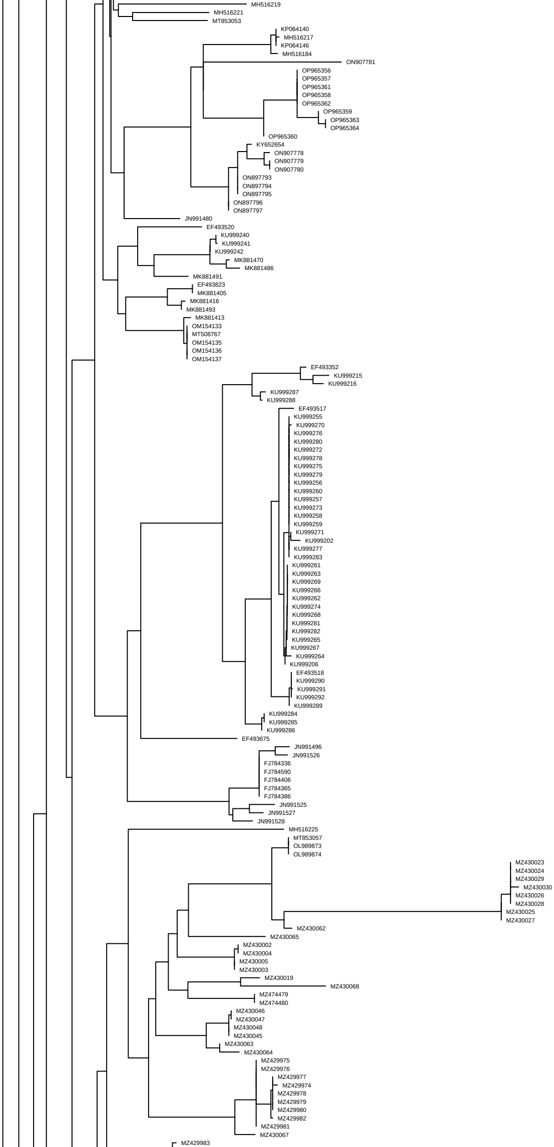
MH516219
MH516221
MT853053
KP064140
MH516217
KP064146
MH516184
ON907781
OP965356
OP965357
OP965361
OP965358
OP965362
OP965359
OP965363
OP965364
OP965360
KY652654
ON907778
ON907779
ON907780
ON897793
ON897794
ON897795
ON897796
ON897797
JN991480
EF493520
KU999240
KU999241
KU999242
MK881470
MK881486
MK881491
EF493823
MK881405
MK881416
MK881493
MK881413
OM154133
MT508767
OM154135
OM154136
OM154137
EF493352
KU999215
KU999216
KU999287
KU999288
EF493517
KU999255
KU999270
KU999276
KU999280
KU999272
KU999278
KU999275
KU999279
KU999256
KU999260
KU999257
KU999273
KU999258
KU999259
KU999271
KU999202
KU999277
KU999283
KU999261
KU999263
KU999269
KU999266
KU999262
KU999274
KU999268
KU999281
KU999282
KU999265
KU999267
KU999264
KU999206
EF493518
KU999290
KU999291
KU999292
KU999289
KU999284
KU999285
KU999286
EF493675
JN991496
JN991526
FJ784336
FJ784590
FJ784406
FJ784365
FJ784386
JN991525
JN991527
JN991528
MH516225
MT853057
OL989873
OL989874
MZ430023
MZ430024
MZ430029
MZ430030
MZ430026
MZ430028
MZ430025
MZ430027
MZ430062
MZ430065
MZ430002
MZ430004
MZ430005
MZ430003
MZ430019
MZ430068
MZ474479
MZ474480
MZ430046
MZ430047
MZ430048
MZ430045
MZ430063
MZ430064
MZ429975
MZ429976
MZ429977
MZ429974
MZ429978
MZ429979
MZ429980
MZ429982
MZ429981
MZ430067
MZ429983

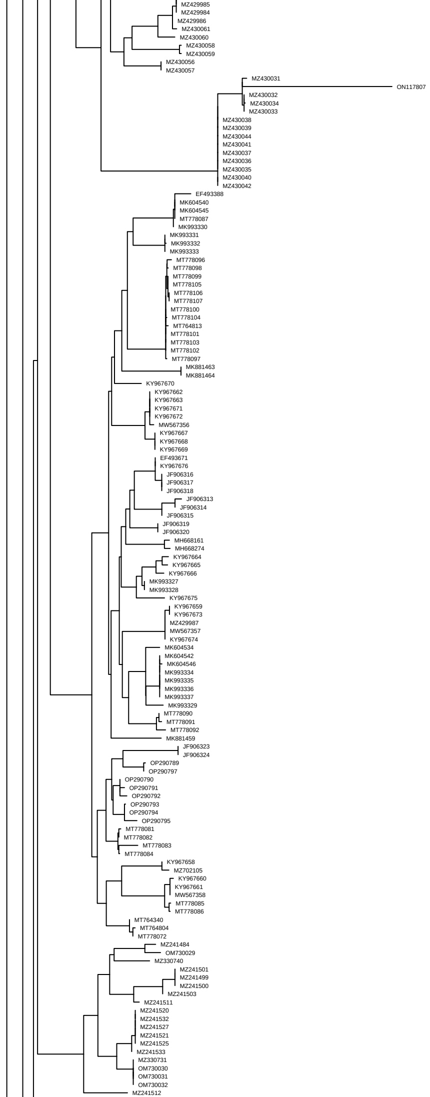
MZ429985
MZ429984
MZ429986
MZ430061
MZ430060
MZ430058
MZ430059
MZ430056
MZ430057
MZ430031
ON117807
MZ430032
MZ430034
MZ430033
MZ430038
MZ430039
MZ430044
MZ430041
MZ430037
MZ430036
MZ430035
MZ430040
MZ430042
EF493388
MK604540
MK604545
MT778087
MK993330
MK993331
MK993332
MK993333
MT778096
MT778098
MT778099
MT778105
MT778106
MT778107
MT778100
MT778104
MT764813
MT778101
MT778103
MT778102
MT778097
MK881463
MK881464
KY967670
KY967662
KY967663
KY967671
KY967672
MW567356
KY967667
KY967668
KY967669
EF493671
KY967676
JF906316
JF906317
JF906318
JF906313
JF906314
JF906315
JF906319
JF906320
MH668161
MH668274
KY967664
KY967665
KY967666
MK993327
MK993328
KY967675
KY967659
KY967673
MZ429987
MW567357
KY967674
MK604534
MK604542
MK604546
MK993334
MK993335
MK993336
MK993337
MK993329
MT778090
MT778091
MT778092
MK881459
JF906323
JF906324
OP290789
OP290797
OP290790
OP290791
OP290792
OP290793
OP290794
OP290795
MT778081
MT778082
MT778083
MT778084
KY967658
MZ702105
KY967660
KY967661
MW567358
MT778085
MT778086
MT764340
MT764804
MT778072
MZ241484
OM730029
MZ330740
MZ241501
MZ241499
MZ241500
MZ241503
MZ241511
MZ241520
MZ241532
MZ241527
MZ241521
MZ241525
MZ241533
MZ330731
OM730030
OM730031
OM730032
MZ241512

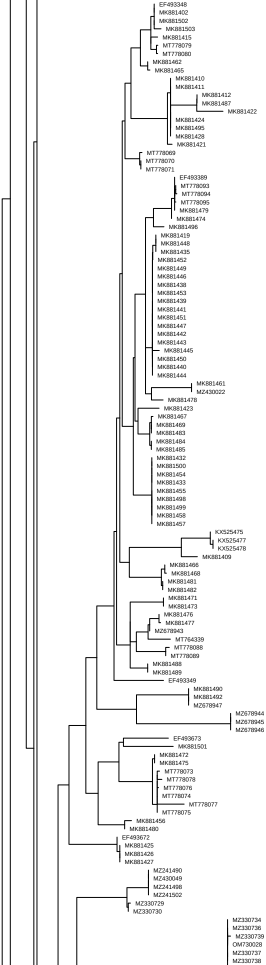
EF493348
MK881402
MK881502
MK881503
MK881415
MT778079
MT778080
MK881462
MK881465
MK881410
MK881411
MK881412
MK881487
MK881422
MK881424
MK881495
MK881428
MK881421
MT778069
MT778070
MT778071
EF493389
MT778093
MT778094
MT778095
MK881479
MK881474
MK881496
MK881419
MK881448
MK881435
MK881452
MK881449
MK881446
MK881438
MK881453
MK881439
MK881441
MK881451
MK881447
MK881442
MK881443
MK881445
MK881450
MK881440
MK881444
MK881461
MZ430022
MK881478
MK881423
MK881467
MK881469
MK881483
MK881484
MK881485
MK881432
MK881500
MK881454
MK881433
MK881455
MK881498
MK881499
MK881458
MK881457
KX525475
KX525477
KX525478
MK881409
MK881466
MK881468
MK881481
MK881482
MK881471
MK881473
MK881476
MK881477
MZ678943
MT764339
MT778088
MT778089
MK881488
MK881489
EF493349
MK881490
MK881492
MZ678947
MZ678944
MZ678945
MZ678946
EF493673
MK881501
MK881472
MK881475
MT778073
MT778078
MT778076
MT778074
MT778077
MT778075
MK881456
MK881480
EF493672
MK881425
MK881426
MK881427
MZ241490
MZ430049
MZ241498
MZ241502
MZ330729
MZ330730
MZ330734
MZ330736
MZ330739
OM730028
MZ330737
MZ330738

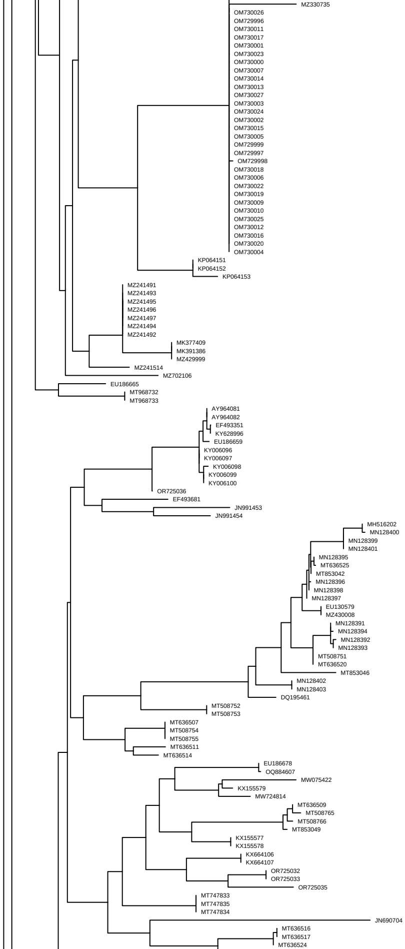
MZ330735
OM730026
OM729996
OM730011
OM730017
OM730001
OM730023
OM730000
OM730007
OM730014
OM730013
OM730027
OM730003
OM730024
OM730002
OM730015
OM730005
OM729999
OM729997
OM729998
OM730018
OM730006
OM730022
OM730019
OM730009
OM730010
OM730025
OM730012
OM730016
OM730020
OM730004
KP064151
KP064152
KP064153
MZ241491
MZ241493
MZ241495
MZ241496
MZ241497
MZ241494
MZ241492
MK377409
MK391386
MZ429999
MZ241514
MZ702106
EU186665
MT968732
MT968733
AY964081
AY964082
EF493351
KY628996
EU186659
KY006096
KY006097
KY006098
KY006099
KY006100
OR725036
EF493681
JN991453
JN991454
MH516202
MN128400
MN128399
MN128401
MN128395
MT636525
MT853042
MN128396
MN128398
MN128397
EU130579
MZ430008
MN128391
MN128394
MN128392
MN128393
MT508751
MT636520
MT853046
MN128402
MN128403
DQ195461
MT508752
MT508753
MT636507
MT508754
MT508755
MT636511
MT636514
EU186678
OQ884607
MW075422
KX155579
MW724814
MT636509
MT508765
MT508766
MT853049
KX155577
KX155578
KX664106
KX664107
OR725032
OR725033
OR725035
MT747833
MT747835
MT747834
JN690704
MT636516
MT636517
MT636524

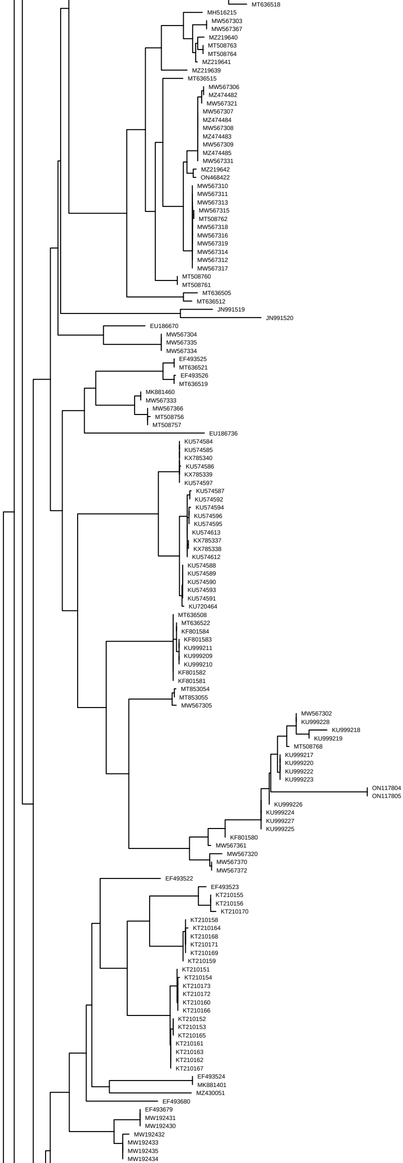

MT636518
MH516215
MW567303
MW567367
MZ219640
MT508763
MT508764
MZ219641
MZ219639
MT636515
MW567306
MZ474482
MW567321
MW567307
MZ474484
MW567308
MZ474483
MW567309
MZ474485
MW567331
MZ219642
ON468422
MW567310
MW567311
MW567313
MW567315
MT508762
MW567318
MW567316
MW567319
MW567314
MW567312
MW567317
MT508760
MT508761
MT636505
MT636512
JN991519
JN991520
EU186670
MW567304
MW567335
MW567334
EF493525
MT636521
EF493526
MT636519
MK881460
MW567333
MW567366
MT508756
MT508757
EU186736
KU574584
KU574585
KX785340
KU574586
KX785339
KU574597
KU574587
KU574592
KU574594
KU574596
KU574595
KU574613
KX785337
KX785338
KU574612
KU574588
KU574589
KU574590
KU574593
KU574591
KU720464
MT636508
MT636522
KF801584
KF801583
KU999211
KU999209
KU999210
KF801582
KF801581
MT853054
MT853055
MW567305
MW567302
KU999228
KU999218
KU999219
MT508768
KU999217
KU999220
KU999222
KU999223
ON117804
ON117805
KU999226
KU999224
KU999227
KU999225
KF801580
MW567361
MW567320
MW567370
MW567372
EF493522
EF493523
KT210155
KT210156
KT210170
KT210158
KT210164
KT210168
KT210171
KT210169
KT210159
KT210151
KT210154
KT210173
KT210172
KT210160
KT210166
KT210152
KT210153
KT210165
KT210161
KT210163
KT210162
KT210167
EF493524
MK881401
MZ430051
EF493680
EF493679
MW192431
MW192430
MW192432
MW192433
MW192435
MW192434

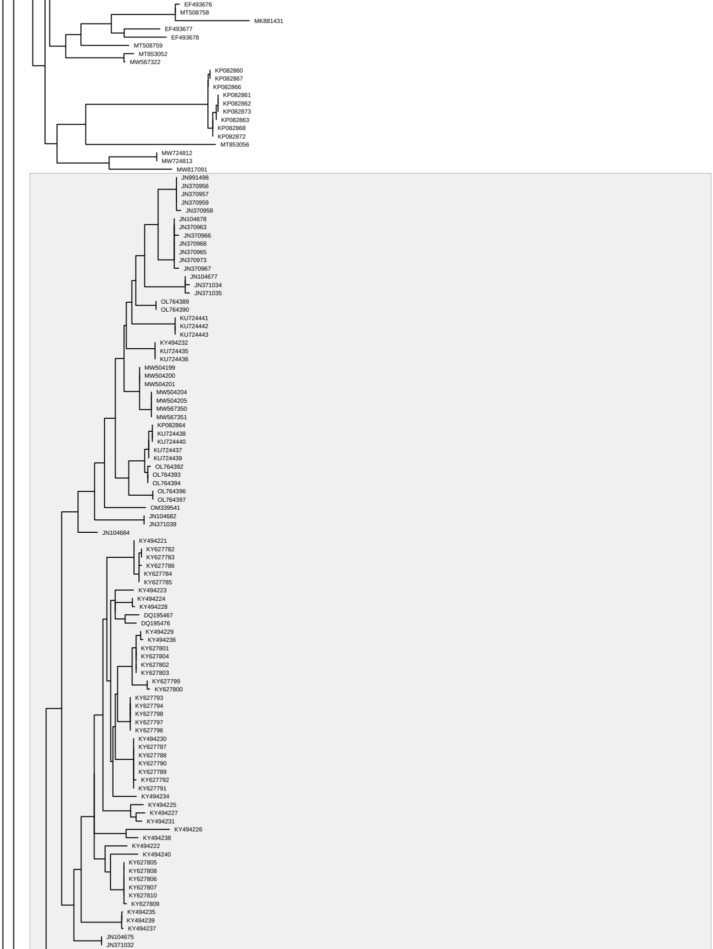
EF493676
MT508758
MK881431
EF493677
EF493678
MT508759
MT853052
MW567322
KP082860
KP082867
KP082866
KP082861
KP082862
KP082873
KP082863
KP082868
KP082872
MT853056
MW724812
MW724813
MW817091
JN991498
JN370956
JN370957
JN370959
JN370958
JN104678
JN370963
JN370966
JN370968
JN370965
JN370973
JN370967
JN104677
JN371034
JN371035
OL764389
OL764390
KU724441
KU724442
KU724443
KY494232
KU724435
KU724436
MW504199
MW504200
MW504201
MW504204
MW504205
MW567350
MW567351
KP082864
KU724438
KU724440
KU724437
KU724439
OL764392
OL764393
OL764394
OL764396
OL764397
OM339541
JN104682
JN371039
JN104684
KY494221
KY627782
KY627783
KY627786
KY627784
KY627785
KY494223
KY494224
KY494228
DQ195467
DQ195476
KY494229
KY494236
KY627801
KY627804
KY627802
KY627803
KY627799
KY627800
KY627793
KY627794
KY627798
KY627797
KY627796
KY494230
KY627787
KY627788
KY627790
KY627789
KY627792
KY627791
KY494234
KY494225
KY494227
KY494231
KY494226
KY494238
KY494222
KY494240
KY627805
KY627808
KY627806
KY627807
KY627810
KY627809
KY494235
KY494239
KY494237
JN104675
JN371032

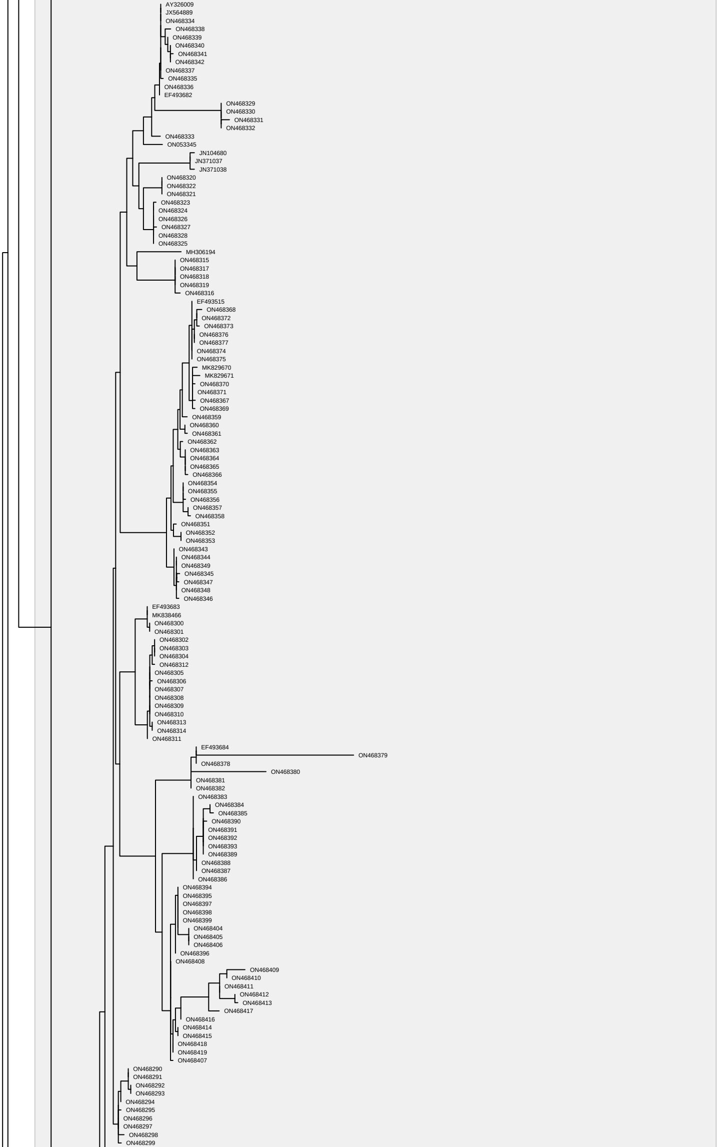
AY326009
JX564889
ON468334
ON468338
ON468339
ON468340
ON468341
ON468342
ON468337
ON468335
ON468336
EF493682
ON468329
ON468330
ON468331
ON468332
ON468333
ON053345
JN104680
JN371037
JN371038
ON468320
ON468322
ON468321
ON468323
ON468324
ON468326
ON468327
ON468328
ON468325
MH306194
ON468315
ON468317
ON468318
ON468319
ON468316
EF493515
ON468368
ON468372
ON468373
ON468376
ON468377
ON468374
ON468375
MK829670
MK829671
ON468370
ON468371
ON468367
ON468369
ON468359
ON468360
ON468361
ON468362
ON468363
ON468364
ON468365
ON468366
ON468354
ON468355
ON468356
ON468357
ON468358
ON468351
ON468352
ON468353
ON468343
ON468344
ON468349
ON468345
ON468347
ON468348
ON468346
EF493683
MK838466
ON468300
ON468301
ON468302
ON468303
ON468304
ON468312
ON468305
ON468306
ON468307
ON468308
ON468309
ON468310
ON468313
ON468314
ON468311
EF493684
ON468379
ON468378
ON468380
ON468381
ON468382
ON468383
ON468384
ON468385
ON468390
ON468391
ON468392
ON468393
ON468389
ON468388
ON468387
ON468386
ON468394
ON468395
ON468397
ON468398
ON468399
ON468404
ON468405
ON468406
ON468396
ON468408
ON468409
ON468410
ON468411
ON468412
ON468413
ON468417
ON468416
ON468414
ON468415
ON468418
ON468419
ON468407
ON468290
ON468291
ON468292
ON468293
ON468294
ON468295
ON468296
ON468297
ON468298
ON468299

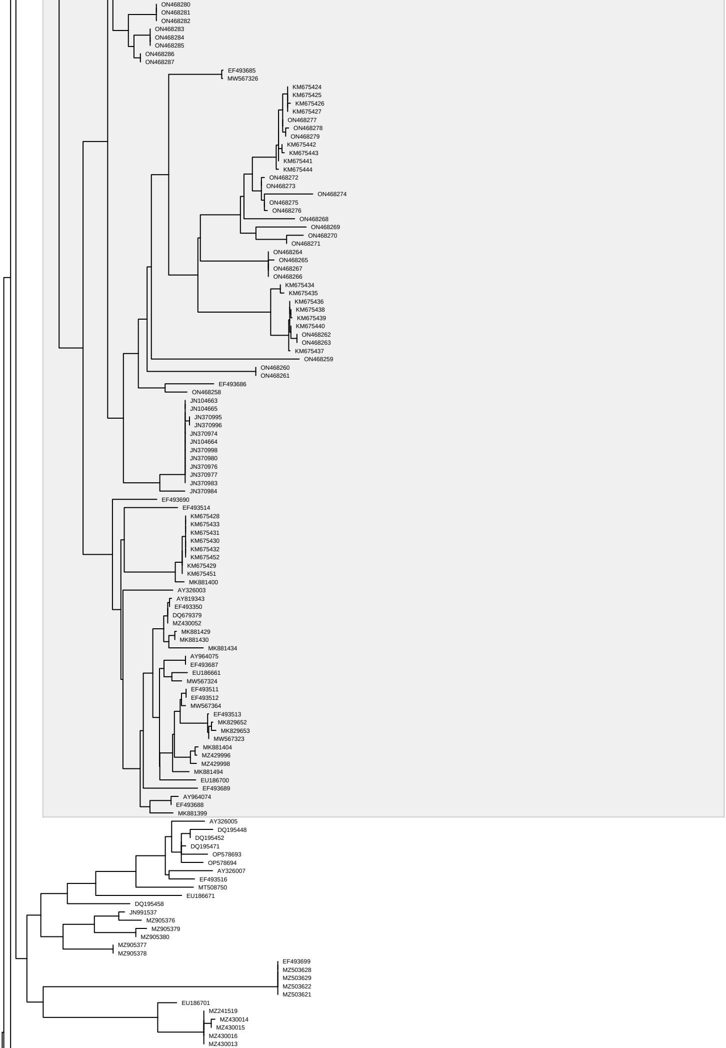
ON468280
ON468281
ON468282
ON468283
ON468284
ON468285
ON468286
ON468287
EF493685
MW567326
KM675424
KM675425
KM675426
KM675427
ON468277
ON468278
ON468279
KM675442
KM675443
KM675441
KM675444
ON468272
ON468273
ON468274
ON468275
ON468276
ON468268
ON468269
ON468270
ON468271
ON468264
ON468265
ON468267
ON468266
KM675434
KM675435
KM675436
KM675438
KM675439
KM675440
ON468262
ON468263
KM675437
ON468259
ON468260
ON468261
EF493686
ON468258
JN104663
JN104665
JN370995
JN370996
JN370974
JN104664
JN370998
JN370980
JN370976
JN370977
JN370983
JN370984
EF493690
EF493514
KM675428
KM675433
KM675431
KM675430
KM675432
KM675452
KM675429
KM675451
MK881400
AY326003
AY819343
EF493350
DQ679379
MZ430052
MK881429
MK881430
MK881434
AY964075
EF493687
EU186661
MW567324
EF493511
EF493512
MW567364
EF493513
MK829652
MK829653
MW567323
MK881404
MZ429996
MZ429998
MK881494
EU186700
EF493689
AY964074
EF493688
MK881399
AY326005
DQ195448
DQ195452
DQ195471
OP578693
OP578694
AY326007
EF493516
MT508750
EU186671
DQ195458
JN991537
MZ905376
MZ905379
MZ905380
MZ905377
MZ905378
EF493699
MZ503628
MZ503629
MZ503622
MZ503621
EU186701
MZ241519
MZ430014
MZ430015
MZ430016
MZ430013

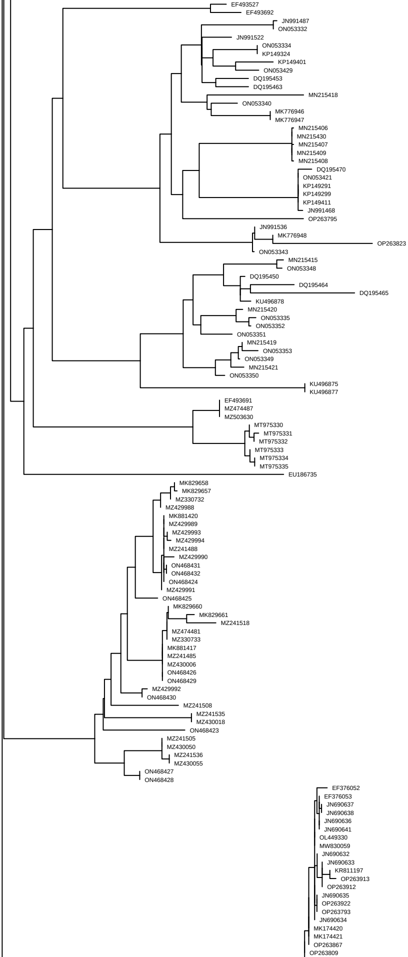
EF493527
EF493692
JN991487
ON053332
JN991522
ON053334
KP149324
KP149401
ON053429
DQ195453
DQ195463
MN215418
ON053340
MK776946
MK776947
MN215406
MN215430
MN215407
MN215409
MN215408
DQ195470
ON053421
KP149291
KP149299
KP149411
JN991468
OP263795
JN991536
MK776948
OP263823
ON053343
MN215415
ON053348
DQ195450
DQ195464
DQ195465
KU496878
MN215420
ON053335
ON053352
ON053351
MN215419
ON053353
ON053349
MN215421
ON053350
KU496875
KU496877
EF493691
MZ474487
MZ503630
MT975330
MT975331
MT975332
MT975333
MT975334
MT975335
EU186735
MK829658
MK829657
MZ330732
MZ429988
MK881420
MZ429989
MZ429993
MZ429994
MZ241488
MZ429990
ON468431
ON468432
ON468424
MZ429991
ON468425
MK829660
MK829661
MZ241518
MZ474481
MZ330733
MK881417
MZ241485
MZ430006
ON468426
ON468429
MZ429992
ON468430
MZ241508
MZ241535
MZ430018
ON468423
MZ241505
MZ430050
MZ241536
MZ430055
ON468427
ON468428
EF376052
EF376053
JN690637
JN690638
JN690636
JN690641
OL449330
MW830059
JN690632
JN690633
KR811197
OP263913
OP263912
JN690635
OP263922
OP263793
JN690634
MK174420
MK174421
OP263867
OP263809

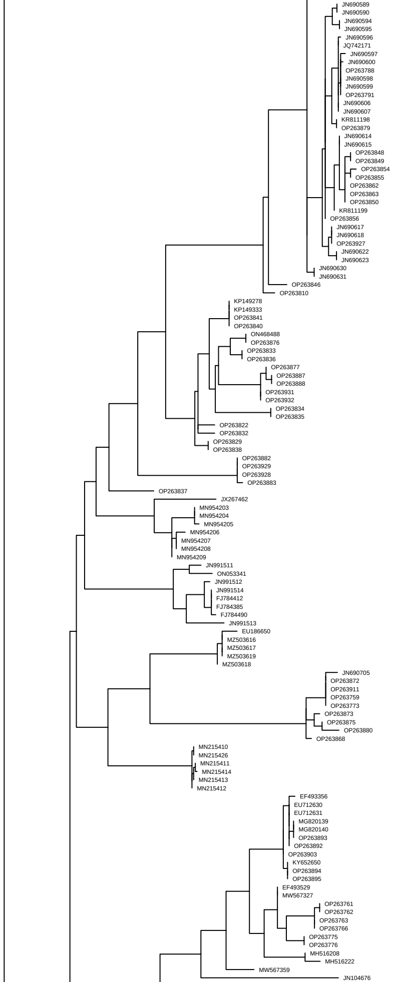
JN690589
JN690590
JN690594
JN690595
JN690596
JQ742171
JN690597
JN690600
OP263788
JN690598
JN690599
OP263791
JN690606
JN690607
KR811198
OP263879
JN690614
JN690615
OP263848
OP263849
OP263854
OP263855
OP263862
OP263863
OP263850
KR811199
OP263856
JN690617
JN690618
OP263927
JN690622
JN690623
JN690630
JN690631
OP263846
OP263810
KP149278
KP149333
OP263841
OP263840
ON468488
OP263876
OP263833
OP263836
OP263877
OP263887
OP263888
OP263931
OP263932
OP263834
OP263835
OP263822
OP263832
OP263829
OP263838
OP263882
OP263929
OP263928
OP263883
OP263837
JX267462
MN954203
MN954204
MN954205
MN954206
MN954207
MN954208
MN954209
JN991511
ON053341
JN991512
JN991514
FJ784412
FJ784385
FJ784490
JN991513
EU186650
MZ503616
MZ503617
MZ503619
MZ503618
JN690705
OP263872
OP263911
OP263759
OP263773
OP263873
OP263875
OP263880
OP263868
MN215410
MN215426
MN215411
MN215414
MN215413
MN215412
EF493356
EU712630
EU712631
MG820139
MG820140
OP263893
OP263892
OP263903
KY652650
OP263894
OP263895
EF493529
MW567327
OP263761
OP263762
OP263763
OP263766
OP263775
OP263776
MH516208
MH516222
MW567359
JN104676

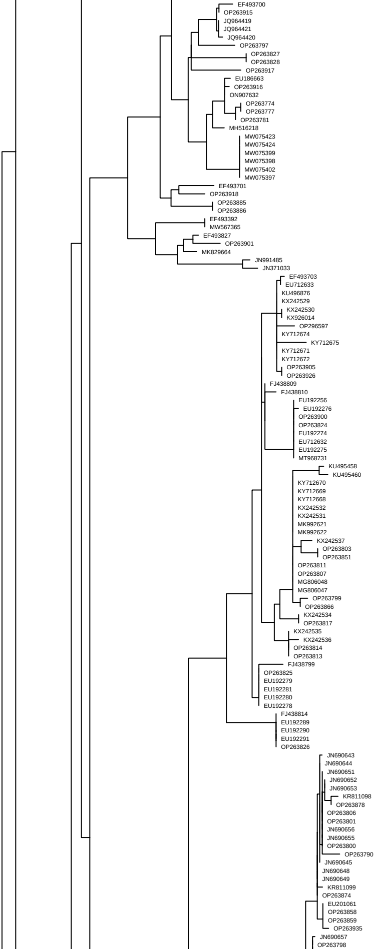
EF493700
OP263915
JQ964419
JQ964421
JQ964420
OP263797
OP263827
OP263828
OP263917
EU186663
OP263916
ON907632
OP263774
OP263777
OP263781
MH516218
MW075423
MW075424
MW075399
MW075398
MW075402
MW075397
EF493701
OP263918
OP263885
OP263886
EF493392
MW567365
EF493827
OP263901
MK829664
JN991485
JN371033
EF493703
EU712633
KU496876
KX242529
KX242530
KX926014
OP296597
KY712674
KY712675
KY712671
KY712672
OP263905
OP263926
FJ438809
FJ438810
EU192256
EU192276
OP263900
OP263824
EU192274
EU712632
EU192275
MT968731
KU495458
KU495460
KY712670
KY712669
KY712668
KX242532
KX242531
MK992621
MK992622
KX242537
OP263803
OP263851
OP263811
OP263807
MG806048
MG806047
OP263799
OP263866
KX242534
OP263817
KX242535
KX242536
OP263814
OP263813
FJ438799
OP263825
EU192279
EU192281
EU192280
EU192278
FJ438814
EU192289
EU192290
EU192291
OP263826
JN690643
JN690644
JN690651
JN690652
JN690653
KR811098
OP263878
OP263806
OP263801
JN690656
JN690655
OP263800
OP263790
JN690645
JN690648
JN690649
KR811099
OP263874
EU201061
OP263858
OP263859
OP263935
JN690657
OP263798

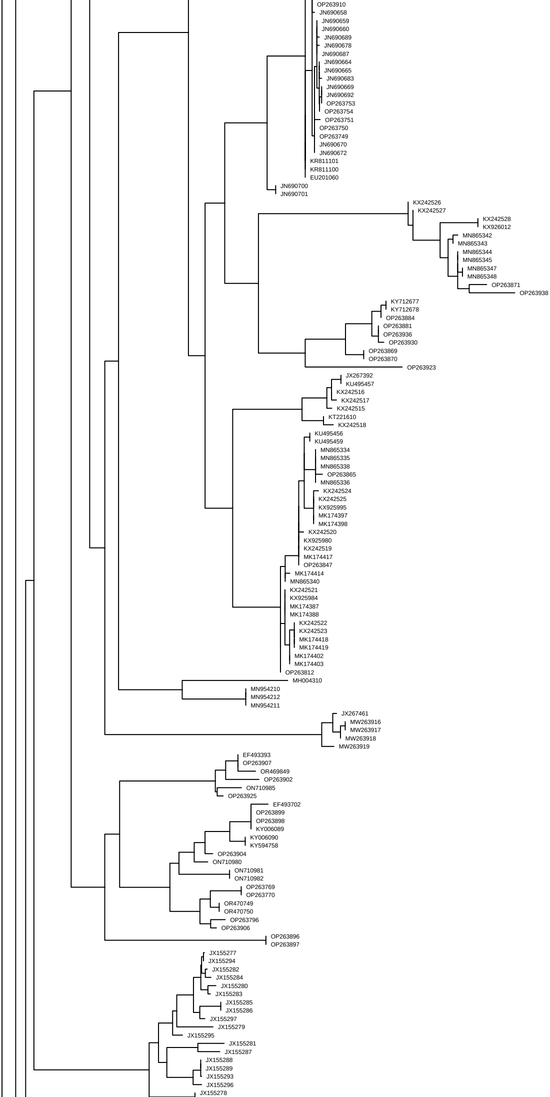
OP263910
JN690658
JN690659
JN690660
JN690689
JN690678
JN690687
JN690664
JN690665
JN690683
JN690669
JN690692
OP263753
OP263754
OP263751
OP263750
OP263749
JN690670
JN690672
KR811101
KR811100
EU201060
JN690700
JN690701
KX242526
KX242527
KX242528
KX926012
MN865342
MN865343
MN865344
MN865345
MN865347
MN865348
OP263871
OP263938
KY712677
KY712678
OP263884
OP263881
OP263936
OP263930
OP263869
OP263870
OP263923
JX267392
KU495457
KX242516
KX242517
KX242515
KT221610
KX242518
KU495456
KU495459
MN865334
MN865335
MN865338
OP263865
MN865336
KX242524
KX242525
KX925995
MK174397
MK174398
KX242520
KX925980
KX242519
MK174417
OP263847
MK174414
MN865340
KX242521
KX925984
MK174387
MK174388
KX242522
KX242523
MK174418
MK174419
MK174402
MK174403
OP263812
MH004310
MN954210
MN954212
MN954211
JX267461
MW263916
MW263917
MW263918
MW263919
EF493393
OP263907
OR469849
OP263902
ON710985
OP263925
EF493702
OP263899
OP263898
KY006089
KY006090
KY594758
OP263904
ON710980
ON710981
ON710982
OP263769
OP263770
OR470749
OR470750
OP263796
OP263906
OP263896
OP263897
JX155277
JX155294
JX155282
JX155284
JX155280
JX155283
JX155285
JX155286
JX155297
JX155279
JX155295
JX155281
JX155287
JX155288
JX155289
JX155293
JX155296
JX155278

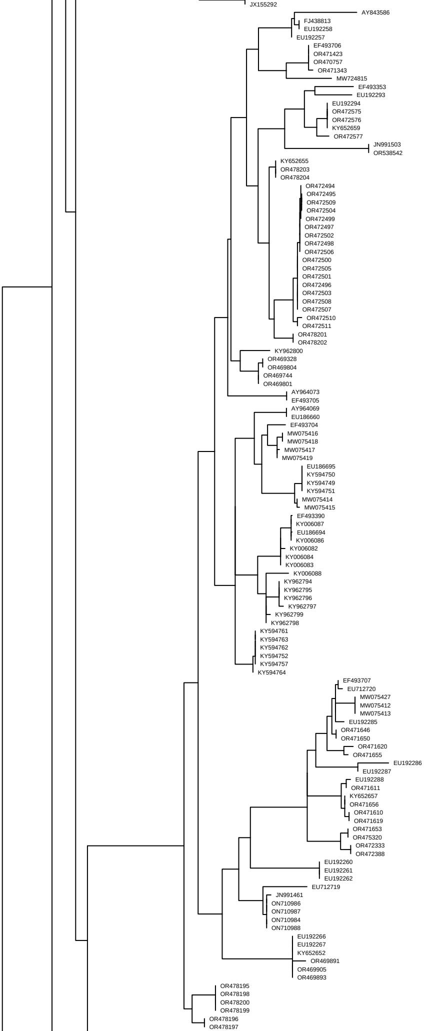

JX155292
AY843586
FJ438813
EU192258
EU192257
EF493706
OR471423
OR470757
OR471343
MW724815
EF493353
EU192293
EU192294
OR472575
OR472576
KY652659
OR472577
JN991503
OR538542
KY652655
OR478203
OR478204
OR472494
OR472495
OR472509
OR472504
OR472499
OR472497
OR472502
OR472498
OR472506
OR472500
OR472505
OR472501
OR472496
OR472503
OR472508
OR472507
OR472510
OR472511
OR478201
OR478202
KY962800
OR469328
OR469804
OR469744
OR469801
AY964073
EF493705
AY964069
EU186660
EF493704
MW075416
MW075418
MW075417
MW075419
EU186695
KY594750
KY594749
KY594751
MW075414
MW075415
EF493390
KY006087
EU186694
KY006086
KY006082
KY006084
KY006083
KY006088
KY962794
KY962795
KY962796
KY962797
KY962799
KY962798
KY594761
KY594763
KY594762
KY594752
KY594757
KY594764
EF493707
EU712720
MW075427
MW075412
MW075413
EU192285
OR471646
OR471650
OR471620
OR471655
EU192286
EU192287
EU192288
OR471611
KY652657
OR471656
OR471610
OR471619
OR471653
OR475320
OR472333
OR472388
EU192260
EU192261
EU192262
EU712719
JN991461
ON710986
ON710987
ON710984
ON710988
EU192266
EU192267
KY652652
OR469891
OR469905
OR469893
OR478195
OR478198
OR478200
OR478199
OR478196
OR478197

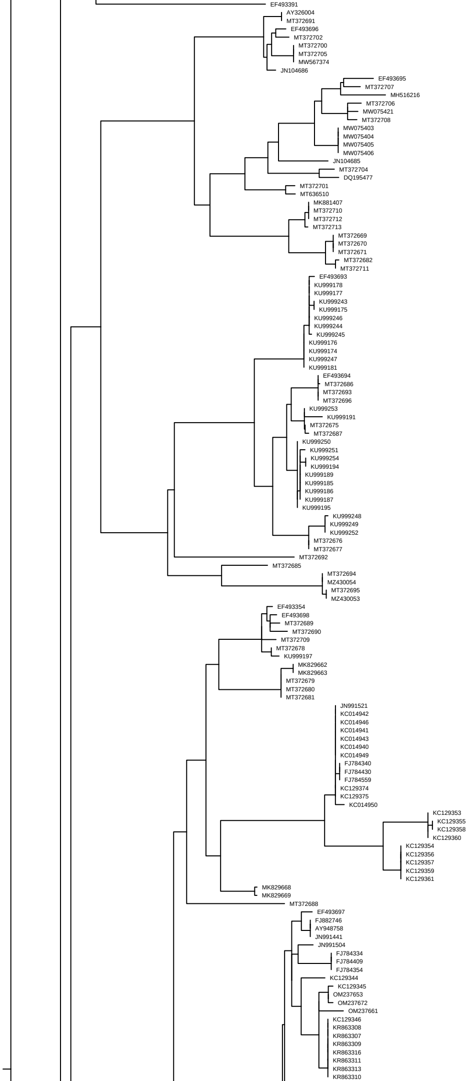
EF493391
AY326004
MT372691
EF493696
MT372702
MT372700
MT372705
MW567374
JN104686
EF493695
MT372707
MH516216
MT372706
MW075421
MT372708
MW075403
MW075404
MW075405
MW075406
JN104685
MT372704
DQ195477
MT372701
MT636510
MK881407
MT372710
MT372712
MT372713
MT372669
MT372670
MT372671
MT372682
MT372711
EF493693
KU999178
KU999177
KU999243
KU999175
KU999246
KU999244
KU999245
KU999176
KU999174
KU999247
KU999181
EF493694
MT372686
MT372693
MT372696
KU999253
KU999191
MT372675
MT372687
KU999250
KU999251
KU999254
KU999194
KU999189
KU999185
KU999186
KU999187
KU999195
KU999248
KU999249
KU999252
MT372676
MT372677
MT372692
MT372685
MT372694
MZ430054
MT372695
MZ430053
EF493354
EF493698
MT372689
MT372690
MT372709
MT372678
KU999197
MK829662
MK829663
MT372679
MT372680
MT372681
JN991521
KC014942
KC014946
KC014941
KC014943
KC014940
KC014949
FJ784340
FJ784430
FJ784559
KC129374
KC129375
KC014950
KC129353
KC129355
KC129358
KC129360
KC129354
KC129356
KC129357
KC129359
KC129361
MK829668
MK829669
MT372688
EF493697
FJ882746
AY948758
JN991441
JN991504
FJ784334
FJ784409
FJ784354
KC129344
KC129345
OM237653
OM237672
OM237661
KC129346
KR863308
KR863307
KR863309
KR863316
KR863311
KR863313
KR863310

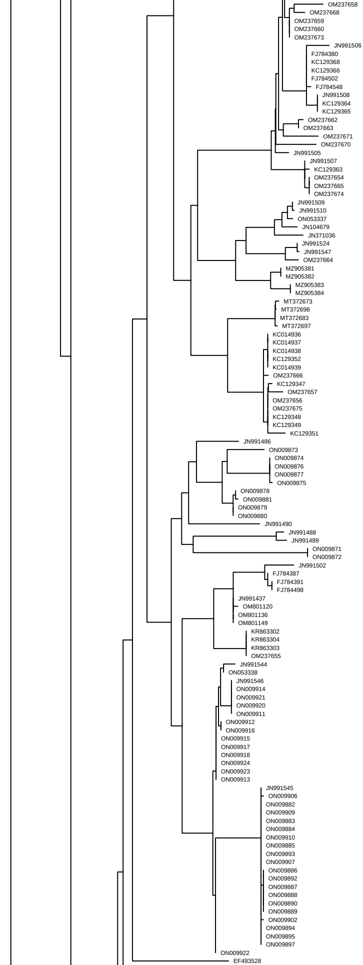
OM237658
OM237668
OM237659
OM237660
OM237673
JN991506
FJ784380
KC129368
KC129366
FJ784502
FJ784548
JN991508
KC129364
KC129365
OM237662
OM237663
OM237671
OM237670
JN991505
JN991507
KC129363
OM237654
OM237665
OM237674
JN991509
JN991510
ON053337
JN104679
JN371036
JN991524
JN991547
OM237664
MZ905381
MZ905382
MZ905383
MZ905384
MT372673
MT372698
MT372683
MT372697
KC014936
KC014937
KC014938
KC129352
KC014939
OM237666
KC129347
OM237657
OM237656
OM237675
KC129348
KC129349
KC129351
JN991486
ON009873
ON009874
ON009876
ON009877
ON009875
ON009878
ON009881
ON009879
ON009880
JN991490
JN991488
JN991489
ON009871
ON009872
JN991502
FJ784387
FJ784391
FJ784498
JN991437
OM801120
OM801136
OM801149
KR863302
KR863304
KR863303
OM237655
JN991544
ON053338
JN991546
ON009914
ON009921
ON009920
ON009911
ON009912
ON009916
ON009915
ON009917
ON009918
ON009924
ON009923
ON009913
JN991545
ON009906
ON009882
ON009909
ON009883
ON009884
ON009910
ON009885
ON009893
ON009907
ON009886
ON009892
ON009887
ON009888
ON009890
ON009889
ON009902
ON009894
ON009895
ON009897
ON009922
EF493528

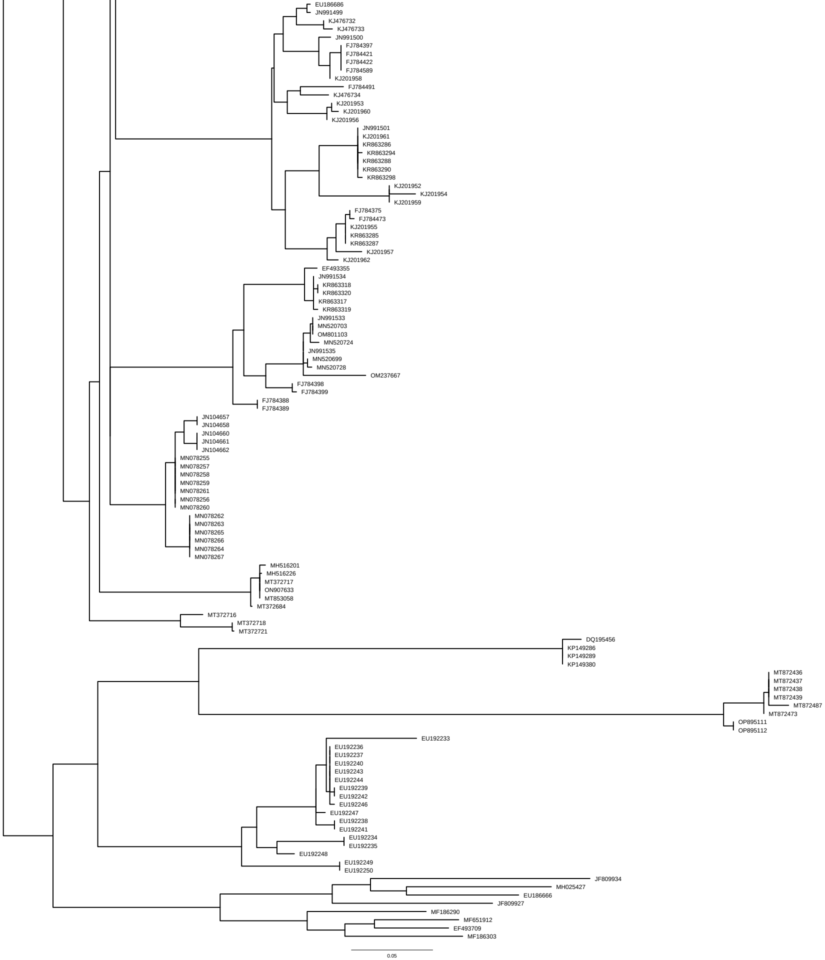
EU186686
JN991499
KJ476732
KJ476733
JN991500
FJ784397
FJ784421
FJ784422
FJ784589
KJ201958
FJ784491
KJ476734
KJ201953
KJ201960
KJ201956
JN991501
KJ201961
KR863286
KR863294
KR863288
KR863290
KR863298
KJ201952
KJ201954
KJ201959
FJ784375
FJ784473
KJ201955
KR863285
KR863287
KJ201957
KJ201962
EF493355
JN991534
KR863318
KR863320
KR863317
KR863319
JN991533
MN520703
OM801103
MN520724
JN991535
MN520699
MN520728
OM237667
FJ784398
FJ784399
FJ784388
FJ784389
JN104657
JN104658
JN104660
JN104661
JN104662
MN078255
MN078257
MN078258
MN078259
MN078261
MN078256
MN078260
MN078262
MN078263
MN078265
MN078266
MN078264
MN078267
MH516201
MH516226
MT372717
ON907633
MT853058
MT372684
MT372716
MT372718
MT372721
DQ195456
KP149286
KP149289
KP149380
MT872436
MT872437
MT872438
MT872439
MT872487
MT872473
OP895111
OP895112
EU192233
EU192236
EU192237
EU192240
EU192243
EU192244
EU192239
EU192242
EU192246
EU192247
EU192238
EU192241
EU192234
EU192235
EU192248
EU192249
EU192250
JF809934
MH025427
EU186666
JF809927
MF186290
MF651912
EF493709
MF186303
0.05
